# Wnt Signaling Prevents the Aβ Oligomer-Induced Mitochondrial Permeability Transition Pore Opening Preserving Mitochondrial Structure in Hippocampal Neurons

**DOI:** 10.1371/journal.pone.0168840

**Published:** 2017-01-06

**Authors:** Macarena S. Arrázola, Eva Ramos-Fernández, Pedro Cisternas, Daniela Ordenes, Nibaldo C. Inestrosa

**Affiliations:** 1 Centro de Envejecimiento y Regeneración (CARE), Departamento de Biología Celular y Molecular, Facultad de Ciencias Biológicas, Pontificia Universidad Católica de Chile, Santiago, Chile; 2 Universidad de Atacama, Facultad de Ciencias Naturales, Departamento de Química y Biología, Copiapó, Chile; 3 Center for Healthy Brain Ageing, School of Psychiatry, Faculty of Medicine, University of New South Wales, Sydney, Australia; 4 Centro de Excelencia en Biomedicina de Magallanes (CEBIMA), Universidad de Magallanes, Punta Arenas, Chile; Georgia Regents University, UNITED STATES

## Abstract

Alzheimer’s disease (AD) is a neurodegenerative disorder mainly known for synaptic impairment and neuronal cell loss, affecting memory processes. Beside these damages, mitochondria have been implicated in the pathogenesis of AD through the induction of the mitochondrial permeability transition pore (mPTP). The mPTP is a non-selective pore that is formed under apoptotic conditions, disturbing mitochondrial structure and thus, neuronal viability. In AD, Aβ oligomers (Aβos) favor the opening of the pore, activating mitochondria-dependent neuronal cell death cascades. The Wnt signaling activated through the ligand Wnt3a has been described as a neuroprotective signaling pathway against amyloid-β (Aβ) peptide toxicity in AD. However, the mechanisms by which Wnt signaling prevents Aβos-induced neuronal cell death are unclear. We proposed here to study whether Wnt signaling protects neurons earlier than the late damages in the progression of the disease, through the preservation of the mitochondrial structure by the mPTP inhibition. To study specific events related to mitochondrial permeabilization we performed live-cell imaging from primary rat hippocampal neurons, and electron microscopy to analyze the mitochondrial morphology and structure. We report here that Wnt3a prevents an Aβos-induced cascade of mitochondrial events that leads to neuronal cell death. This cascade involves (a) mPTP opening, (b) mitochondrial swelling, (c) mitochondrial membrane potential loss and (d) cytochrome *c* release, thus leading to neuronal cell death. Furthermore, our results suggest that the activation of the Wnt signaling prevents mPTP opening by two possible mechanisms, which involve the inhibition of mitochondrial GSK-3β and/or the modulation of mitochondrial hexokinase II levels and activity. This study suggests a possible new approach for the treatment of AD from a mitochondrial perspective, and will also open new lines of study in the field of Wnt signaling in neuroprotection.

## Introduction

Alzheimer’s disease (AD) is a neurodegenerative disorder characterized by memory loss and cognitive decline [1]. The main reason for the neuronal dysfunction in AD is the amyloid-β peptide, specifically the oligomers (Aβos), which are the most neurotoxic species [2–4]. Even though synaptic failure and neuronal death are classical features of AD, defects in mitochondria have been detected earlier [5,6]. The Aβ peptide acts within the mitochondria, affecting mitochondrial structure by favoring the opening of the mitochondrial permeability transition pore (mPTP) [7]. The composition of the mPTP is not completely clear yet, but several proteins have been described as part of the pore conformation, such as the voltage-dependent calcium channel (VDAC), the adenine nucleotide translocase (ANT), cyclophilin D (CypD) [8,9], the F-ATP synthase [10,11], proteins from the Bcl-family as Bax [12], and others. In AD, Aβos facilitate the interaction of CypD with the other components to form an open and irreversible conformation of the pore [13]. The induction of the mPTP permeates the mitochondrial inner membrane (IMM), facilitating the exchange of solutes between the mitochondrial matrix and the cytoplasm, thereby producing a phenomenon known as mitochondrial swelling. Mitochondrial swelling occurs along with several mitochondrial perturbations, including multiple cellular stresses as ROS generation, calcium deregulation, mitochondrial membrane potential collapse, and the release of pro-apoptotic factors into the cytoplasm, such as cytochrome *c*. These mitochondrial perturbations, known as the “mitochondrial cascade hypothesis in AD”, precede the synaptic damage, the neuronal cell death, and the deficits in learning/memory ability in AD [6,14].

Previous studies have demonstrated that impaired Wnt signaling pathway is related with aging [15] and Aβ-induced neurotoxicity [16,17]. The Wnt pathway is activated by the binding of a Wnt ligand to its Frizzled (Fz) receptor and to the co-receptor LRP6 [18], which inhibits cytosolic glycogen synthase kinase-3β (GSK-3β) via phosphorylation at serine 9 and causes β-catenin to accumulate in the cytoplasm and ultimately translocate to the nucleus, where it regulates the expression of Wnt target genes [19,20]. The activation of the Wnt signaling pathway displays neuroprotective properties in AD [21–23], by protecting hippocampal neurons from Aβ-induced synaptic failures and cell death *in vitro* [24,25] and rescuing from behavioral impairment in AD mice models [17,26,27]. These alterations are considered late events in the progression of the disease, however, whether Wnt signaling pathway protects at the initial steps of the “mitochondrial cascade in AD”, thus preventing the late damage, remains so far unexplored.

We report here that activation of Wnt signaling with the ligand Wnt3a prevents mitochondrial membrane permeabilization by inhibiting mPTP opening in hippocampal neurons exposed to Aβos. In addition, Wnt3a preserves mitochondrial morphology, the integrity of mitochondrial membranes, as evidenced by its inhibition of mitochondrial membrane potential dissipation and cytochrome *c* release, thus protecting neuronal viability. Our results suggest that the mPTP inhibition observed in response to Wnt signaling activation is mediated by the inhibition of GSK-3β via 2 possible mechanisms, namely via modulation of the mitochondrial detachment/translocation process of hexokinase II (HKII) and via the interaction of phosphorylated GSK-3β with ANT in the mPTP protein complex. These results suggest that Wnt signaling prevents neuronal cell death by protecting the mitochondrial structure and inhibiting mitochondrial permeabilization.

## Materials and Methods

### Animals

Animals were born and maintained at the Animal Facility of the Pontificia Universidad Católica de Chile under sanitary barrier in ventilated racks and in closed colonies. Experimental procedures were approved by the Bioethical and Biosafety Committee of the Faculty of Biological Sciences of the university. Euthanasia were performed using 5–8% isofluorane. Pregnant Sprague-Dawley rats (E18) were used to prepare primary hippocampal neurons in culture as was described previously [24]. For more details, see S1 File.

### Formation of amyloid-β oligomers

Synthetic Aβ_1–42_ peptides corresponding to wild-type human Aβ were obtained from Genemed Synthesis, Inc. (San Francisco, CA). An Aβ peptide stock solution was prepared by dissolving freeze-dried aliquots of Aβ in 1,1,1,3,3,3-hexafluoro-2-propanol (HFIP, Sigma H-8508) at 1 mM. For oligomer preparation, the peptide film was dissolved in dimethyl sulfoxide (DMSO, Sigma D2650) at 5 mM and was then diluted in PBS to a final concentration of 100 μM. The preparation was incubated overnight to allow Aβos formation and then centrifuged (14,000 rpm, 1 h, 4°C) to eliminate any formed fibril [28]. An aliquot of Aβos solution is used to quantify protein with the Qubit 2.0 Fluorometer (Invitrogen, Carlsbad, CA, USA) in order to determine the final concentration (70–80 μM). Aβos were visualized by electron microscopy and analyzed by Tris-Tricine SDS gel electrophoresis, as previously described [29,30].

### Aβo treatments in cultured hippocampal neurons and brain slices

For *in vitro* treatments, freshly prepared Aβos were diluted to 5 μM in Neurobasal medium without supplements and were directly added to the cultured hippocampal neurons, which were chronically stimulated for 24 h to conduct neuronal viability assays. For live-cell imaging experiments, neurons were acutely exposed to 10 μM Aβos for 7 min. Hippocampal slice preparations were treated for 1 h with 5 μM Aβos diluted in artificial cerebrospinal fluid (ACSF). For all treatments, vehicle solution corresponds to a PBS/DMSO solution prepared by using the same volume of DMSO used for the Aβos preparation, and then diluted in Neurobasal or ACSF, as appropriate.

### Calcein/cobalt imaging for mPTP opening assay

For *in vivo* cell imaging, neurons were seeded on 25-mm cover slips at a density of 1.5 x 10^5^ cells per cover slip. mPTP opening was measured using the Image-iT ^™^ LIVE Mitochondrial Transition Pore Assay Kit (I35103) (Molecular Probes, Carlsbad, CA) with modifications, as described in previous studies [31,32]. At 10 days *in vitro* (DIV), hippocampal neurons were treated with recombinant mouse Wnt3a (300 ng/ml, 24 h) (R&D Systems, Inc., MM, cat n° 1324-WN/CF) or Neurobasal control medium and were loaded with the labeling mix solution (4 μM calcein-AM, 50 nM MitoTracker Orange, 1 mM CoCl_2_ and 1 μM Hoechst 33342 dye in Neurobasal medium) for 30 min at 37°C. Cultures were washed with the recording solution, Tyrode’s buffer (135 mM NaCl, 5 mM KCl, 1.8 mM CaCl_2_, 1 mM MgCl_2_, 10 mM HEPES, 5.6 mM glucose, pH 7.3) supplemented with 1mM CoCl_2_ and imaged on an Olympus DSU IX81 spinning-disk confocal microscope in the same conditions. The excitation/emission (ex/em) peaks of calcein after hydrolysis occur at 494/517 nm. The cytoplasmic signal of calcein is quenched by cobalt ions, which do not affect the mitochondrial signal. As a result of stimulating mPTP opening, including the permeabilization of the IMM, calcein is released from the mitochondrial matrix, resulting in the redistribution and rapid decay of mitochondrial calcein fluorescence [32]. After the basal signals were measured, 10 μM Aβos was added at 3 min, and fluorescence was recorded for 10 min. Hoechst staining (**λ**ex/em, 350/461 nm) was not used during the experiment but was used to determine the viability of the neurons before and after Aβos exposure. The mitochondrial pattern of calcein staining was verified using MitoTracker as a mitochondrial marker, and mitochondria that were initially positive for both labels were used for the analysis. The ionophore ionomycin (0.5 μM), which induces Ca^2+^ overload, was used as a positive control for mPTP induction. Pre-incubation of neurons with cyclosporin A (CsA, 20 μM) for 30 min was used to inhibit pore opening [33,34]. Wnt inhibitors were used as follow: sFRP2 (250 nM) was pre-incubated with the recombinant Wnt3a for 30 min and then applied together to the neurons for 24 h; DKK1 (100 ng/ml) was added for 30 min to the neurons and then co-incubated with the Wnt3a ligand for 24 h; ICG001 (20μM) was co-incubated with Wnt3a for 24 h; and 6-BIO (10nM) was used in the same conditions as Wnt3a, for 24 h. For calcium-induced mitochondrial swelling experiments, cells were treated with control or recombinant Wnt3a, as described above, and washed twice with Neurobasal medium. In order to measure the mPTP opening, neurons were subsequently loaded with calcein/Co^2+^ as was explained before. The measurement of mitochondrial buffering ability after calcium application was done permeabilizing plasma membrane of the hippocampal neurons with 9 μM digitonin for 2 min in an intracellular solution (130 mM K-gluconate, 7m M KCl, 4m M ATP-Mg^2+^, 0.3 mM GTP-Na, 10 mM HEPES, 10 mM phosphocreatinedi(Tris), 0.4% biocytin, pH 7.4, 0.308 Osmol/Kg). In these conditions we achieved only the plasma membrane permeabilization without affecting the mitochondria [35]. The recording solution used for the calcium-induced mPTP assay was the intracellular solution supplemented with 1mM CoCl_2_. After 100 s recording the basal signal, we applied 20μM CaCl_2_ and after 300 s 100μM CaCl_2_ was applied. Time-lapse images were acquired on an Olympus DSU IX81 spinning-disk confocal microscope in the same conditions explained before. The images were analyzed using Stack-T-function/DeltaF plugin from NIH ImageJ software.

### Electron microscopy

Hippocampal slices were used for electron microscopy analysis according to standard procedures. Ultra-thin sections were examined using a Phillips Tecnai 12 transmission electron microscope at 80 kV at the Electron Microscope Facility of the Faculty of Biological Sciences, Pontificia Universidad Católica de Chile, Santiago, Chile. For each treatment, 40 to 50 digital images at 16,500X magnification were obtained, and they were manually analyzed with ImageJ in a blinded fashion by measuring the mitochondrial area [32,36]. Ultrastructural features of the mitochondria, such as the integrity of the membrane and cristae, were determined for each mitochondrion concomitantly with the measurement of morphological parameters [37]. Membranes or cristae were considered to be intact when the entire structure was preserved and organized and the mitochondria appeared normal, as previously described [38]. Quantitative analysis was performed with n = 3 using GraphPad Prism 5.01 software. For more details, see S1 File.

### 3-Dimensional (3D) image reconstruction

The mitochondrial network was 3-dimensionally reconstructed as previously described [39] from mito-Cherry-transfected neurons. To overexpress mito-Cherry, we used NeuroMag (OZ Bioscience, Marseille, France) according to the manufacturer's protocol. Briefly, neurons after 10 DIV at 70,000 cells per well were incubated with the mito-Cherry plasmid and NeuroMag beads as previously described [40]. After 48 h, the neurons were used in treatments. To visualize mitochondria, neurons were illuminated at 563 nm. Serial images (z-stacks) were collected with a Nikon Eclipse C2si spectral confocal microscope with a z-step of 0.35 μm. Z-projection and 3D isosurface reconstructions of the mitochondrial network and individual mitochondria were performed with a manually adjusted threshold level of 76.4582 using Imaris 5.7.0 (Bitplane Inc., Saint Paul, MN) at the Microscope Facility of the University of Concepción, Chile. Mitochondrial volume was calculated based on 3D isosurface reconstructions, as previously described [37,41]. In each condition, 6 to 8 neurons were analyzed, with n = 4 independent experiments.

### Mitochondrial membrane potential, _m_ΔΨ

Time-lapse experiments to detect _m_**Δ**Ψ were performed using hippocampal neurons loaded with 50 nM of the fluorescent probe MitoTracker-Orange (Molecular Probes) for 30 min at 37°C [29,30,39]. MitoTracker-Orange fluoresces according to the _m_**Δ**Ψ. Fluorescence variations were analyzed in the same manner as the calcein/cobalt measurements, using an Olympus DSU IX81 spinning-disk confocal microscope with λex/em values of 554/576 nm [42].

### Cytochrome-c release

Neurons treated with 5 μM Aβos in the presence or absence of Wnt3a for 24 h were loaded with MitoTracker Orange (50 nM), fixed and then analysed by immunofluorescence using a mouse anti-cytochrome-c antibody (BD Pharmingen, San Diego, CA) to detect cytochrome *c* localization. Hoechst staining was used to detect live cells and only those neurons with no condensed nucleus were considered for the analyses. Images were captured with an Olympus FluoView1000 Confocal Microscope and analyzed using NIH ImageJ software. Manders’ Coefficient M2 was calculated to determine the colocalization of cytochrome *c* with mitochondria.

### Neuronal cell viability

Hippocampal neurons plated on poly-L-lysine-coated coverslips (30,000 neurons/coverslip) were treated under different conditions for 24 h. Live and dead neurons (+calcein/-EthD1 and -calcein/+EthD1, respectively) were analyzed in non-fixed cells with the LIVE/DEAD Viability/Cytotoxicity Kit for mammalian cells (Molecular Probes) [43]. Apoptotic nuclei were also analyzed in fixed cells after treatment by using Hoechst 33342 stain (1 μg/ml in distilled water) (Molecular Probes) as previously described [29,30].

### Western blot

Mitochondria were isolated from hippocampal slices using the Mitochondrial Isolation Kit for Tissue (Pierce Biotechnology, Rockford, IL) according to the manufacturer's instructions. Following isolation, mitochondria were lysed in 2% CHAPS in Tris-buffered saline (25 mM Tris, 0.15 M NaCl, pH 7.2). The samples (30 μg) were then subjected to electrophoresis on 15% SDS-polyacrylamide gels. We used rabbit anti-phosphorylated (Ser9) GSK-3β (1:1000) (Cell Signaling Technology Inc., Danvers, MA), rabbit anti-GSK-3β (1:1000) (Santa Cruz Biotechnoloy, Inc., Santa Cruz, CA), mouse anti- Cytochrome C (1:1000) (Abcam, Cambridge MA), goat anti-HKII N-19 (1:1000) (Santa Cruz Biotechnology, Inc., Santa Cruz, CA), mouse anti-GAPDH (1:5000) (Santa Cruz Biotechnology) as a cytoplasmic loading control, and rabbit anti-COXIV antibody (1:5000) (Cell Signaling Technology) as a loading control for the mitochondrial fraction.

### Co-immunoprecipitation assay

Protein extracts were obtained from hippocampal slices lysed in RIPA buffer (50 mM Tris, 150 mM NaCl, 1 mM EGTA, 1 mM EDTA, 0.5% deoxycholate, 1% NP-40, and 0.1% SDS) that was supplemented with protease (Halt protease inhibitor cocktail, Thermo Fisher Scientific Inc., Rockford, IL) and phosphatase inhibitors (in mM: 0.33 Na_2_P_2_O_7_, 1 Na_3_VO_4_, and 50 NaF). Immunoprecipitation was performed using a mouse anti-phosphorylated (Ser9) GSK-3β antibody (1 μg) (Santa Cruz Biotechnology) and protein G-agarose. Immunocomplexes were separated via SDS-PAGE, transferred to PVDF membranes (Thermo Fisher Scientific) and immunoblotted with a rabbit anti-phosphorylated (Ser9) GSK-3β antibody (1:1000) (Cell Signaling Technology) to estimate the efficiency of the assay. Interactions of different proteins with p-GSK-3β were evaluated using a rabbit anti-ANT1-4 antibody (1:500) (Santa Cruz Biotechnology) and a rabbit anti-CypD antibody (1:1000) (Thermo Fisher Scientific). Rabbit anti-GSK-3β (1:1000) and mouse anti-GAPDH (1:5000) antibodies were used as positive and negative controls, respectively, for interaction with p-GSK-3β.

### Hexokinase activity

Hippocampal slices were prepared as described above and treated with recombinant Wnt3a (300 ng/ml), DKK1 (30 ng/mL), rWnt3a+DKK1, and 2-deoxy-D-glucose (2-DG, 7 mM; a competitive inhibitor of HK) [44] and analyzed for hexokinase activity (HK) after 3 h of treatment. Then, the slices were washed with ACSF and subjected to mechanical disaggregation and centrifuged at 500 g for 5 min at 4°C. The tissue was resuspended in isolation medium (250 mM sucrose, 20 mM HEPES, 10 mM KCl, 1.5 mM MgCl_2_, 1 mM EDTA, 1 mM DTT, 2 mg/mL aprotinin, 1 mg/mL pepstatin A, and 2 mg/mL leupeptin) at a 1:3 dilution, sonicated at 4°C, and then centrifuged at 1,500 g for 5 min at 4°C. Finally, the HK activity of the supernatant was quantified. For the assay, the purified fraction was mixed with the reaction medium (25 mM Tris-HCl, 1 mM DTT, 0.5 mM NADP/Na^+^, 2 mM MgCl_2_, 1 mM ATP, 2 U/mL G6PDH, and 10 mM glucose), and the mixture was incubated at 37°C for 30 min. The reaction was stopped by the addition of 10% trichloroacetic acid, and the generation of NADPH was measured at 340 nm [45].

### Quantification and statistical analysis

The data are shown as the mean and standard error of the mean (SEM) from 3–8 independent experiments for live-cell imaging, each with n = 3–4 replicates. Replicates (n = 3–4) were also used for immunofluorescence, mitochondrial network reconstruction and electron microscopy analysis. *p* values were obtained using two-way ANOVA for grouped data and one-way ANOVA for bar graphs, and the *post hoc* Bonferroni correction was also used. Error bars indicate SEM. *p<0.05; **p<0.005; ***p<0.0005.

## Results

### Wnt3a prevents Aβo-induced mitochondrial permeability transition pore opening

**Fig 1:**
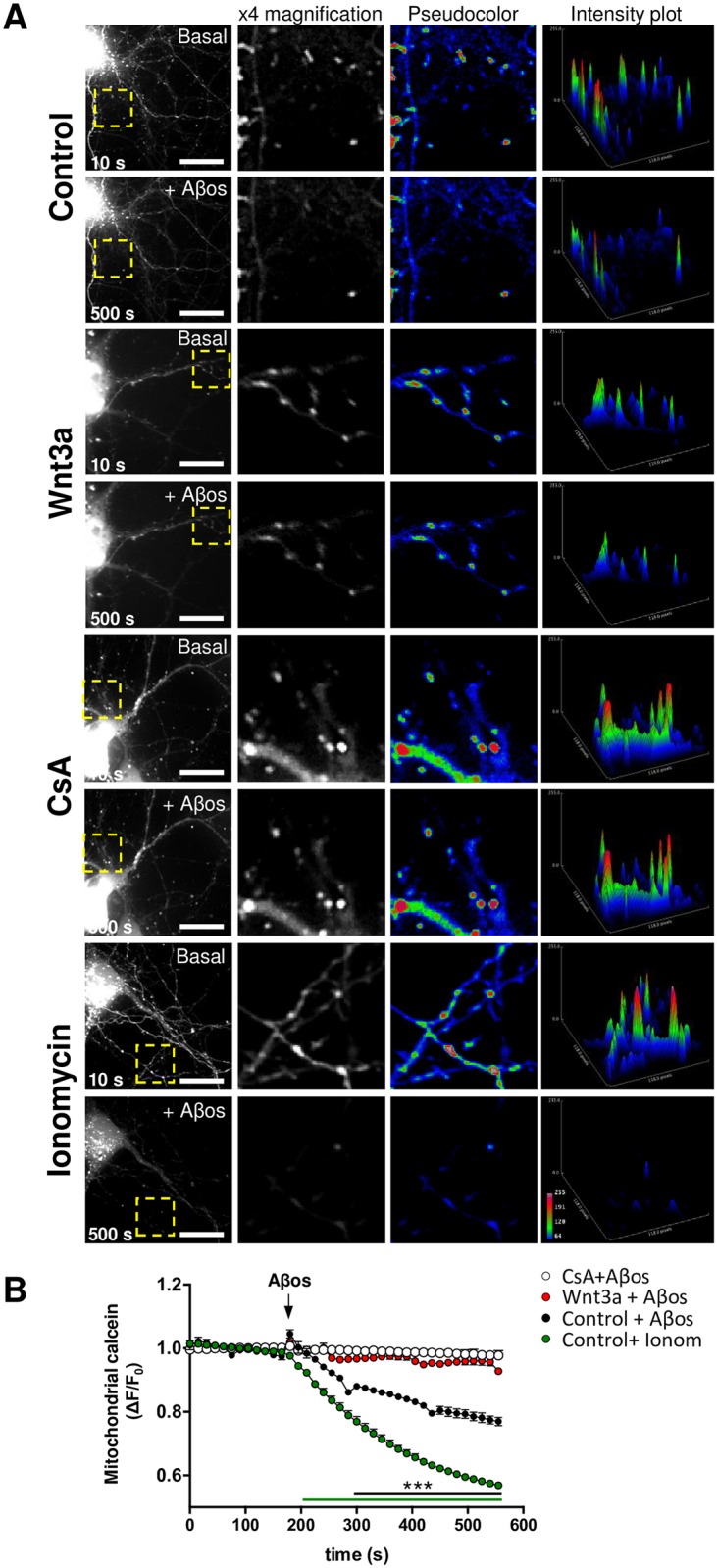
Wnt signaling prevents Aβo-induced mitochondrial permeability transition pore opening in living neurons. (A) Representative images showing DIV10 hippocampal neurons treated with control media or with recombinant Wnt3a protein (300 ng/ml) for 24 h and loaded with calcein-Co^2+^ to stain mitochondria. 20 μM CsA and 0.5 μM ionomycin were used as negative and positive controls for mPTP induction, respectively. The fluorescence intensity decay of mitochondrial calcein corresponds to mPTP opening in response to Aβos exposure. Yellow rectangles indicate magnified regions. Scale bar, 10 μm. Close-up photos are shown as 8-bit black and white images (x4 magnification) as well as pseudocoloured images. Intensity plots represent the fluorescence intensity profile of each magnified region. (B) Time-lapse quantification of the fluorescence intensity variations. The white horizontal bar on the graph indicates the addition of Aβos. The results represent the analysis of 5–7 neurites from 3–4 neurons per experiment. The graph shows the mean ± SEM of n = 7 independent experiments. Statistical analysis was performed with two-way ANOVA with *post hoc* Bonferroni correction: ***p<0.0005.

To directly monitor the mPTP opening in hippocampal neurons *in vitro* we used a live-cell imaging assay, based on the labeling of mitochondria with calcein-Co^2+^ [31,32]. Wnt3a ligand was used to induce the activation of the Wnt signaling pathway in hippocampal neurons, as its neuroprotective properties against Aβ and different apoptotic insults are well described [24,46]. The mPTP opening was stimulated with Aβos, and as a result of permeabilization of the mitochondrial inner membrane the mitochondrial calcein fluorescence rapidly decayed in control neurons, whereas Wnt3a-treated neurons did not show any obvious changes in response to Aβos (Fig 1A). Quantification showed significant differences between control and Wnt3a-treated neurons in response to Aβos stimulation (Fig 1B). Ionomycin was used to completely induce mitochondrial permeabilization; and Cyclosporin A (CsA), a specific inhibitor of mPTP [33], to inhibit Aβos-induced pore opening. CsA treatment showed the same protective effect as Wnt3a on mPTP inhibition, based on the absence of significant differences between the two treatments after Aβos exposure (Fig 1B, compare the white and red curves), revealing the specificity of the effect of Wnt3a on mPTP inhibition, which will be confirmed hereinafter.

**Fig 2:**
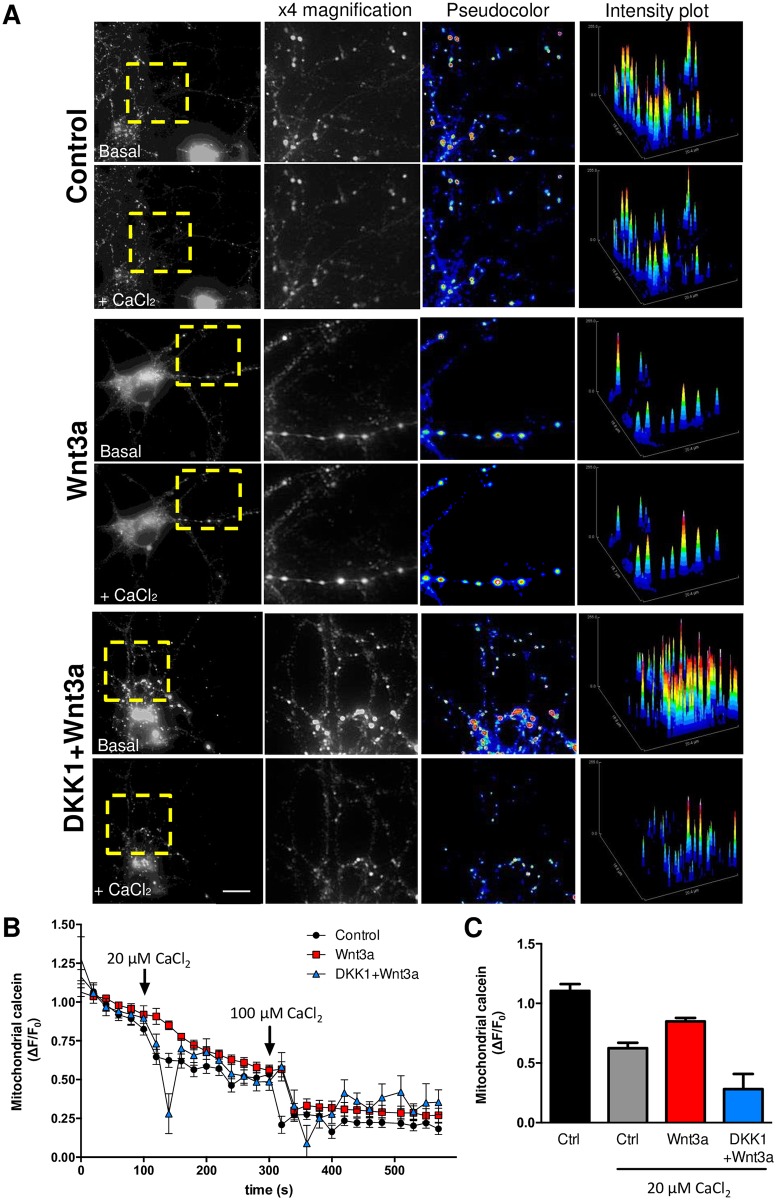
Resistance to calcium-induced mPTP opening in response to Wnt3a. (A) Representative images showing DIV10 hippocampal neurons treated with control media, recombinant Wnt3a protein (300 ng/ml) or pretreated 30 min with DKK1 (100 ng/mL) and coincubated with Wnt3a for 24 h and loaded with calcein-Co^2+^ to stain mitochondria. The fluorescence intensity decay of mitochondrial calcein corresponds to mPTP opening in response to 20 μM CaCl_2_ exposure. Yellow rectangles indicate magnified regions. Scale bar, 8 μm. Close-up images are shown in black and white (x4 magnification) as well as pseudocoloured images. Intensity plots represent the fluorescence intensity profile of each magnified region. (B) Time-lapse quantification of the fluorescence intensity variations. The arrows indicate the addition of 20 and 100 μM CaCl_2_. The results represent the analysis of 5–10 neurites from 2 neurons per experiment. (C) The graph represents the measurements of each condition after the addition of 20 μM CaCl_2_ (140 s) of the experiment, normalized to the basal average registered previous to the stimulus. mPTP opening is visualized as a decay in the fluorescence. The graph shows the mean ± SEM of n = 3–5 independent experiments. Statistical analysis was performed using one-way ANOVA *p<0.05.

In order to verify the potential effect of Wnt3a on mPTP inhibition, we evaluated whether Wnt signaling activation render mitochondria more resistant to calcium-induced mPTP, as has been described before using the same assay in intact cells [47] (Fig 2). Mitochondrial calcein intensity transiently decreased in response to calcium pulses (20 μM and 100 μM) in control neurons, immediately returning to basal levels after each pulse (Fig 2B). This transient response was less in Wnt3a-treated cells in response to the 20μM CaCl_2_, indicating that Wnt signaling activation render mitochondria more resistance to calcium-induced mPTP (Fig 2). The protective effect produced by Wnt3a was inhibited when neurons were preincubated with DKK1 (Fig 2C), a well characterized Wnt signaling inhibitor [48]. These results suggest that Wnt3a protects mitochondria from mPTP induction and confirm the results above-mentioned in Fig 1.

### Classical mitochondrial morphological changes in Aβ-induced permeability transition are prevented by Wnt signaling

**Fig 3:**
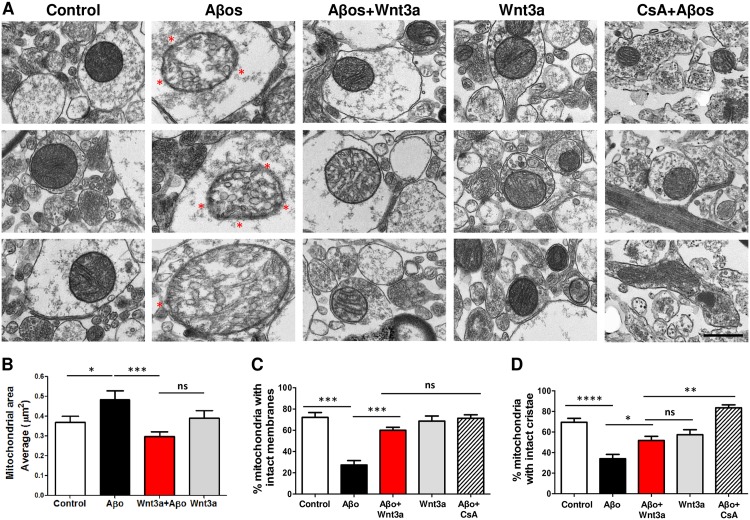
Wnt3a protects mitochondria from morphological and structural alterations induced by Aβos. Hippocampal slices (400 μm) were pre-incubated for 4 h with recombinant Wnt3a and then treated with 5 μM Aβos for 1 h. The slices pre-treated with CsA (20 μM, 30 min) were maintained in ACSF for 4 h (to keep the same experimental conditions as Wnt3a-treated slices) until Aβos treatment. (A) Electron micrographs show three representative mitochondria for each treatment. The red asterisk indicates specific regions with disrupted mitochondrial membrane in the Aβos group. Crista disorganization is also clearly observed in this group. Scale bar, 500 nm. (B) Morphological analysis of mitochondria shows the average area of mitochondria in each condition. (C) Ultrastructural analysis of mitochondrial membrane integrity indicates the percentage of mitochondria that exhibit intact membranes. (D) The organization of mitochondrial cristae is represented in a graph showing the percentage of mitochondria with intact cristae. Statistical analysis was performed on data from three independent slices by using one-way ANOVA and *post hoc* Bonferroni correction: *p< 0.05, **p<0.005, ***p<0.0005.

Mitochondria undergo several morphological changes through the mPTP induction, including mitochondrial swelling. To assess these alterations we measured the mitochondrial area using electron microscopy [32], and two integrity parameters: the membrane disruption and the cristae disorganization. The analysis was performed on the CA1 region of the hippocampus and specifically focused on mitochondria at the synaptic contacts between CA3 axons and CA1 dendrites from the stratum radiatum zone (S1A Fig). Hippocampal slices were acutely treated with Wnt3a for 4h and then co-incubated with Aβos for 1h, enough time to produce mitochondrial damage but not neuronal death (S2 Fig). Only Aβos-treated slices showed enlarged and swollen mitochondria compared with control slices. Wnt3a treatment prevented these morphological changes, and mitochondria with or without Aβos had the same normal shape as under control conditions (Fig 3A and S1B Fig). Compared with the control group, the mean mitochondrial area value in the Aβos group increased by 31.1% (Fig 3B). The increase in the mean value for the Aβos group reflected an augmentation in the size of a subset of mitochondria in the upper 50th percentile rather than an upward size shift of the entire population. This result is demonstrated in Table 1 by the 37.1% and 104.2% increases in the 75th percentile and in the maximum values, respectively, compared with the negligible changes in the minimum values and the 10.2% increase in the 25th-percentile values when the control and Aβos groups are compared. Similar results were obtained for mitochondrial diameter and perimeter (not shown). By contrast, mitochondria from the Wnt3a+Aβos group were structurally similar to mitochondria from the control group; they showed non-significant changes compared with control mitochondria (Fig 3B, Table 1). No changes in mitochondrial biomass were observed with any of the treatments by the measurement of the number of mitochondria per area (S1C Fig), indicating no effects on mitochondrial biogenesis and ruling out mitochondrial fragmentation which should lead to an increase in the number of mitochondria.

**Table 1:** Percentile values and statistical analysis of mitochondrial area based on electron microscopy.

Group	Control	Aβos	Aβos+Wnt3a	Wnt3a
**n =**	113	118	152	76
**Minimum**	0.015	0.033	0.025	0.079
**25th Percentile**	0.127	0.14	0.1343	0.1795
		***(+10*.*2)***^a^	***(+5*.*7)***^a^	***(+41*.*3)***^a^
**Median**	0.214	0.2545	0.1915	0.2395
		***(+18*.*9)***^a^	***(-10*.*5)***^a^	***(+11*.*9)***^a^
**75th Percentile**	0.542	0.743	0.3465	0.559
		***(+37*.*1)***^a^	***(-36*.*1)***^a^	***(+3*.*1)***^a^
**Maximum**	1.571	3.209	2.481	1.523
		***(+104*.*2)***^a^	***(+57*.*9)***^a^	***(-3*.*0)***^a^
**Mean**	0.3682	0.4828	0.2978	0.3906
		***(+31*.*1)***^a^	***(-19*.*1)***^a^	***(+6*.*1)***^a^
**SD**	0.3344	0.4922	0.2976	0.324
**SEM**	0.03146	0.04531	0.02414	0.03716

The table shows a percentile-grouped analysis for each treatment. The values are represented in square micrometers (μm^2^);

^“a”^ indicates the percentage increase in each group value compared with the corresponding control group.

An ultrastructural analysis of each mitochondrion per group showed a clear deterioration of both the mitochondrial membranes and the cristae in hippocampal slices treated with Aβos (Fig 3A). Vesicular cristae and vesicular swollen mitochondria were also observed in Aβos-exposed slices (Fig 3A, red asterisk); these are features of the mitochondrial morphological changes that occur during apoptotic processes [38]. The percentage of mitochondria with intact membranes and cristae significantly decreased in Aβos-treated slices (44.8 ± 8.1% and 35.5 ± 6.1% decrease in membrane and cristae integrity, respectively) compared with control samples, whereas slices treated with Wnt3a+Aβos did not show significant changes from the control (Fig 3C and 3D). CsA was used as a control to inhibit Aβo-induced mPTP and to assess the mitochondrial structure in this condition. CsA protection against Aβos reached similar levels to those observed in hippocampal slices treated with Wnt3a+Aβos, specifically in the analysis of the membranes (Fig 3B; hatched bar), and confirmed the *in vitro* results obtained above in the mPTP assay (Fig 1B).

These results suggest that the activation of Wnt signaling prevents the size changes during mitochondrial swelling triggered in Aβos-induced mPTP opening and protects mitochondria from permeabilization and cristae disorganization, favoring the maintenance of mitochondrial integrity.

**Fig 4:**
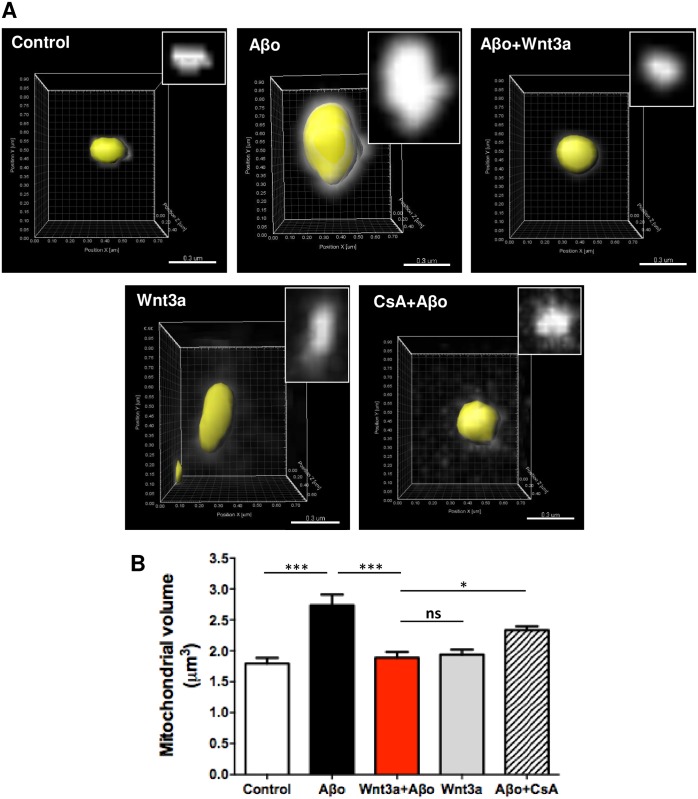
Mitochondrial volume variation in response to Aβos and Wnt3a. Neurons transfected with mito-Cherry at DIV10 were treated for 24 h with 5 μM Aβos and Wnt3a or were pre-treated for 30 min with 20 μM CsA and then exposed to Aβos. Images show individual 3D-reconstructed mitochondria. Scale bar, 0.3 μm. The graph represents the measurement of mitochondrial volume performed with the Imaris software and used for the mitochondrial network reconstruction. The results are shown as the mean of n = 4 independent experiments and the statistical analysis was performed using one-way ANOVA and *post hoc* Bonferroni correction: *p<0.05, ***p<0.0005.

Due to the fact that mitochondrial swelling involves changes in the volume of the organelle, we performed 3D reconstructions of individual mitochondria from hippocampal neurons (Fig 4A), as has been previously described [41]. Aβos induced a significant increase in the average volume of mitochondria, which was prevented by Wnt3a to reach control levels (Fig 4B). Together, these findings indicate that the morphological changes that occur in Aβo-exposed mitochondria correspond to mitochondrial swelling. Furthermore, the Wnt3a-protective effect on mitochondrial morphology is directly associated with its ability to inhibit the mPTP opening, as was described above in Fig 1.

### Integrity-dependent functions of mitochondria are affected during mPTP opening and also prevented by Wnt signaling

**Fig 5:**
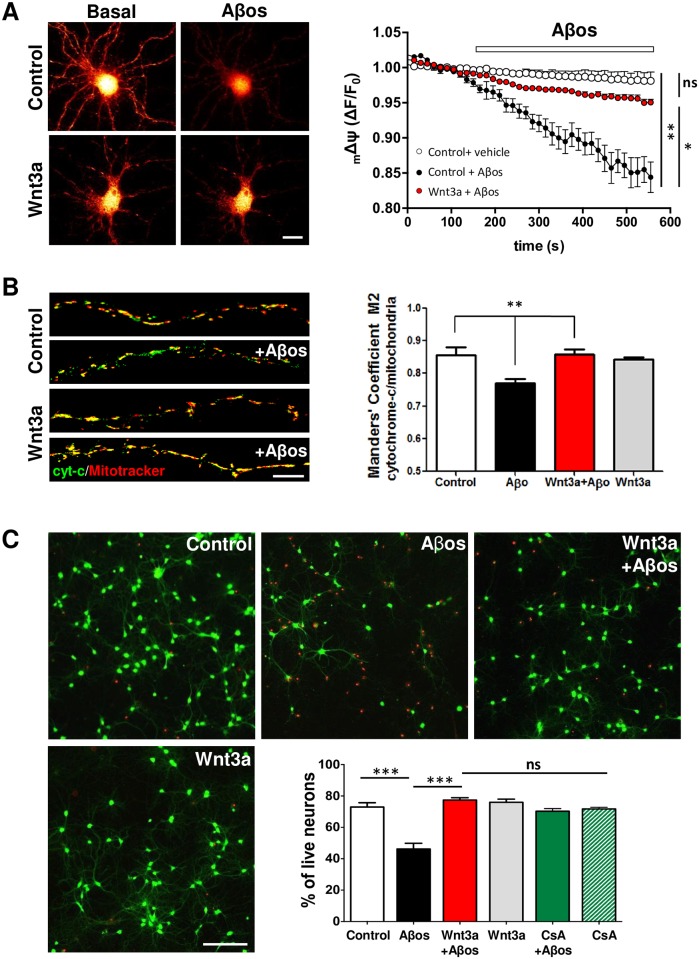
Wnt3a-mediated mPTP inhibition also prevents mitochondrial membrane potential (_m_ΔΨ) collapse, cytochrome-c release, and cell death. (A) _m_ΔΨ, DIV10 hippocampal neurons were treated with control media or with Wnt3a protein for 24 h and were loaded with MitoTracker. Representative red pseudocolored neurons from images obtained before (10 s) and after (500 s) Aβo exposure. The graph represents the time-lapse quantification in neurites for each condition and shows the mean ± SEM of 5 independent experiments. Scale bar, 10 μm. (B) cytochrome-c release, immunodetection of cytochrome-c in neurons treated with 5 μM Aβos in the presence or absence of Wnt3a for 24 h and loaded with MitoTracker-Orange (50 nM). Representative neurites show the colocalization between cytochrome *c* (green) and the mitochondrial marker (red). Manders’ Coefficient M2 was calculated to determine the colocalization of cytochrome *c* with mitochondria. Quantification represents the results of three independent experiments, with 10–15 neurons analyzed per experiment. Scale bar, 5 μm. (C) Live/Dead assay of neurons treated with Wnt3a+Aβos for 24 h or with CsA (20 μM) for 30 min before Aβo treatment. Neurons loaded with calcein/EthD1 were analyzed by epifluorescence microscopy to detect neuronal viability. Neurons stained in green (positive for calcein) represent live cells, whereas the red nuclei correspond to dead cells. The graph shows the percentages of live neurons (calcein/EthD1 ratio) under different treatment conditions. Scale bar, 100 μM. The measurements represent the results of 6 independent experiments. Statistical analysis was performed using one-way ANOVA and *post hoc* Bonferroni correction: *p<0.05; **p<0.005; ***p<0.0005.

The induction of the mPTP facilitates the disruption of the mitochondrial membranes, thereby affecting the mitochondrial membrane potential (_m_ΔΨ). As a consequence, mitochondria release apoptotic factors that trigger neuronal cell death cascades [49]. Incubation of control neurons with Aβos induced a significant loss of the _m_ΔΨ. No statistical differences were observed between control and Wnt3a neurons in the presence of Aβos (Fig 5A). In addition, we evaluated cytochrome *c* release from mitochondria in response to Aβos and Wnt3a treatments. Biochemical analyses did not show any differences in neither mitochondrial nor cytoplasmic cytochrome *c* levels in response to any treatment (not shown). However, when cytochrome *c* is specifically measured in the neurites of cultured neurons by immunofluorescence, we observe a slight but significant effect on cytochrome *c* release from mitochondria in Aβos treated neurons. Manders’ Coefficient M2 was used to analyse the overlapping of the cytochrome *c* labeling with the mitochondria. A significant decrease in the M2 coefficient was observed in neurites evaluated from Aβos-treated neurons, which was prevented by Wnt3a incubation reaching control levels (Fig 5B). In these conditions we also observed a significant decrease in the percentage of live neurons (+calcein/-EthD1) in the Aβos treatment compared with controls (44.09 ± 3.09% vs. 72.97 ± 2.74%, respectively) (Fig 5C). Neurons that were co-incubated with Aβos+Wnt3a did not show significant differences from control neurons (77.40 ± 1.49% vs. 72.97 ± 2.74%, respectively). The same results were observed by counting apoptotic nuclei by Hoechst staining (S3 Fig). CsA treatment was used to determine whether this Wnt3a-mediated protection is related to the inhibition of permeability transition. Neurons that were co-incubated with Aβos and CsA did not show significant differences compared with control or Wnt3a+Aβos-treated neurons (Aβos+CsA: 70.26 ± 1.71) (Fig 5C), suggesting that both Wnt3a and CsA protect neurons by the same mechanism, the inhibition of the mPTP. These results suggest a novel mechanism by which the Wnt signaling pathway might protect neurons from Aβos-induced cell death.

### The effect of Wnt3a on mPTP inhibition is not dependent on the transcription of Wnt target genes

**Fig 6:**
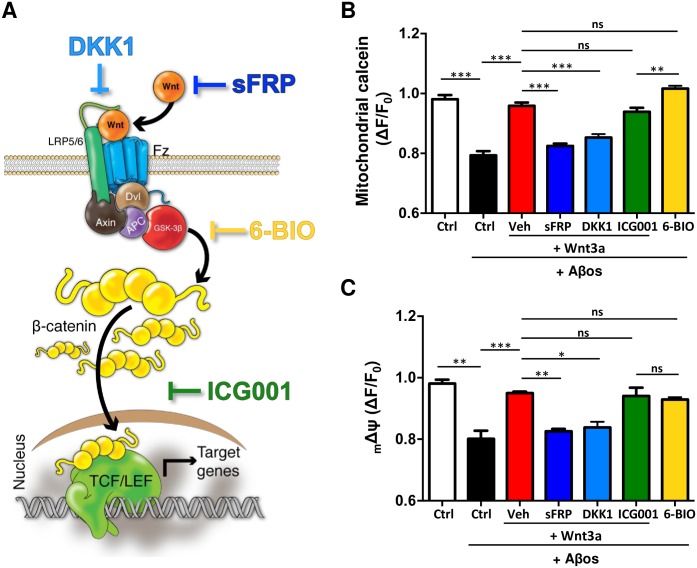
The inhibition of Wnt target genes transcription does not abolish the protective effect of Wnt3a on mPTP opening. (A) Wnt signaling cascade representing the mechanism of action of different Wnt antagonists and inhibitors: sFRP2 binds to Wnt ligand preventing the binding with the receptor; DKK1 binds to the co-receptor LRP6; ICG001 down-regulates β-catenin/T cell factor signaling; and 6-BIO is a specific inhibitor of GSK-3β, thereby it activates the Wnt signaling. (B) mPTP opening assay performed in the presence of Wnt inhibitors: sFRP2 (250 nM), DKK1 (100 ng/mL), ICG001 (20 μM) and 6-BIO (10 nM). The graph represents the measurements of each condition at the end point (500 s) of the experiment, normalized to the basal average registered previous to Aβos addition. mPTP opening is visualized as a decay in the fluorescence. (C) _m_ΔΨ was evaluated in the same conditions described in (B). Statistical analysis was performed using one-way ANOVA *post hoc* Bonferroni correction: *p<0.05; **p<0.005; ***p<0.0005. n = 3–7 independent experiments.

In order to determine the mechanism by which Wnt signaling pathway inhibits the mPTP, we blocked Wnt signaling in different steps (Fig 6A) and we evaluated their effects with the mPTP opening assay. The first step of the signaling, that is, the binding of the ligand with the Fz receptor, was blocked using two classical antagonists: sFRP2, which binds to the Wnt ligand; and DKK1, which blocks the receptor complex [48]. Both inhibitors significantly decreased the protective effect of Wnt3a over Aβos-induced mPTP opening (Fig 6B). The ICG001, an inhibitor of Wnt/β-catenin-dependent transcription [50] was not able to prevent Wnt3a-protection (Fig 6B), which suggests that the protective effect of Wnt3a on mPTP opening is not dependent on the transcription of Wnt target genes. By contrast, the direct inhibition of GSK-3β, one of the main components of the “destruction complex” of β-catenin, with 6-bromoindirubin-3′-oxime (6-BIO) [30], showed the same protective effect as Wnt3a against Aβos (Fig 6B), suggesting that this kinase could be involved in the protection that Wnt signaling produces on mitochondria. Same results were observed with all the inhibitors when the _m_ΔΨ was evaluated (Fig 6C).

### Wnt3a increases mitochondrial phosphorylated GSK-3β: Possible mechanisms for Wnt-dependent mPTP regulation

**Fig 7:**
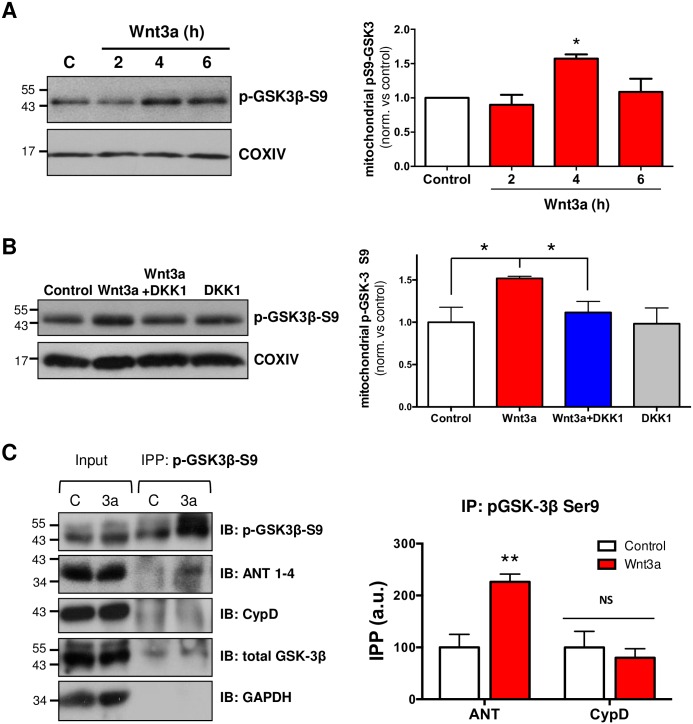
Activation of Wnt signaling modulates mitochondrial GSK-3β phosphorylation and p-GSK-3β/ANT interaction. Hippocampal slices were treated with DKK1 (100 ng/mL) for 30 min before and during Wnt3a treatment. (A) Western blot analysis of mitochondrial fractions obtained from hippocampal slices shows p-GSK3β-S9 levels in mitochondria in response to 300 ng/mL of Wnt3a. The graph shows a significant increase in mitochondrial p-GSK3β-S9 after 4 h of Wnt3a treatment. (B) Inhibition of Wnt3a-mediated induction of mitochondrial p-GSK3β-S9 by DKK1 (100 ng/mL) at 4 h of co-treatmet (n = 3). The graphs represent the densitometric analysis of p-GSK3β-S9 in mitochondrial fractions. Protein levels were normalized to COXIV. (C) Immunoprecipitation assay with phosphorylated-GSK3β-S9 from hippocampal slices treated with Wnt3a for 4 h. *Input* corresponds to whole-slice lysate, and *IPP*: *p-GSK3β-S9* corresponds to the fraction immunoprecipitated with a phosphorylated GSK3β-S9 antibody. Immunoblotting (IB) was performed to detect p-GSK3β-S9, ANT and CypD. Total GSK-3β and GAPDH were used as positive and negative controls, respectively, for the immunoprecipitation assay. Lane C: control; 3a: recombinant Wnt3a. The densitometric analysis was performed from 4 independent experiments. Statistical analyses were conducted using one-way ANOVA and *post hoc* Bonferroni correction: *p<0.05; **p<0.005.

Mitochondrial GSK-3β has been described as a trigger of the permeability transition [51]. The inhibition of GSK-3β through serine 9 phosphorylation is associated with cardioprotection and with the regulation of kidney and liver injury through the inhibition of mPTP opening [52–54]. Interestingly, mPTP-related GSK-3β phosphorylation occurs in mitochondria at the same residue (Ser9) that leads to Wnt signaling activation in the intracellular compartment [55]. To study GSK-3β behavior in brain mitochondria, we isolated mitochondria from hippocampal slices. We found that Wnt3a triggered the accumulation of phosphorylated GSK-3β (p-GSK3β-S9) in the mitochondrial fraction, with a peak of GSK-3β inactivation after 4 h of treatment (Fig 7A). To evaluate the specificity of this effect, we inhibited the activation of Wnt signaling using DKK1. The accumulation of p-GSK3β-S9 in the mitochondrial fraction was significantly prevented when Wnt signaling was inhibited, even in the presence of the Wnt3a ligand (Fig 7B).

To evaluate the possibility that the Wnt3a-induced mitochondrial p-GSK3β-S9 might also interact with some of the protein components of the mPTP, as has been previously described in other models [56], we performed immunoprecipitation assays for p-GSK3β-S9 with ANT and CypD. Wnt signaling induced an increase in the association between p-GSK3β-S9 and ANT, whereas no significant changes were observed with CypD (Fig 7C).

**Fig 8:**
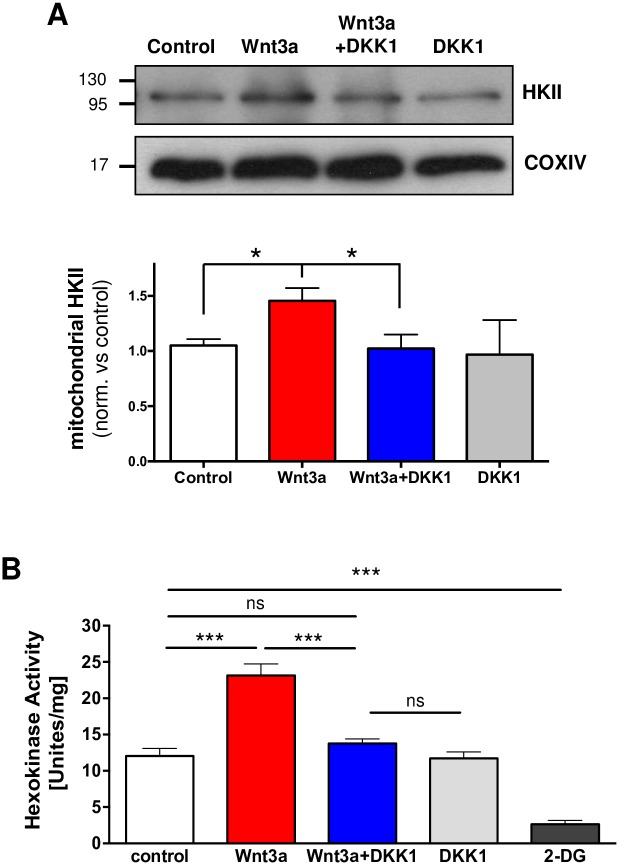
Wnt3a increments mitochondrial HKII levels and HK kinase activity. Hippocampal slices were treated with DKK1 (100 ng/mL) for 30 min before and during Wnt3a treatment. (A) Western blot analysis of mitochondrial fractions shows increase HKII levels induced by Wnt3a (300 ng/mL) and the inhibition produced by DKK1 (100 ng/mL) at 4 h of co-treatmet (n = 3). Graphs represent the densitometric analysis of and HKII levels in mitochondrial fractions. Protein levels were normalized to COXIV. (B) HK activity was measured in hippocampal slices treated with recombinant Wnt3a for 3 h. DKK1 was used to inhibit the effect of Wnt3a, and the competitive inhibitor of HK, 2-DG (7 mM), was used as an assay control to inhibit basal HK activity. Statistical analyses were conducted using one-way ANOVA and *post hoc* Bonferroni correction: *p<0.05; ***p<0.0005.

Another regulator of mPTP opening is the mitochondrial hexokinase II (HKII), which stabilizes the mPTP in its closed conformation; HKII detachment from mitochondria propagates a conformational change that leads to pore opening [57]. Interestingly, GSK-3β activity has been shown to regulate the release of HKII from mitochondria in response to Aβ, thereby enhancing susceptibility to cell death through the mitochondrial permeability transition [58]. To correlate both events we tested Wnt3a effect over mitochondrial HKII levels. A significant increase of HKII in mitochondrial fractions was observed in Wnt3a-treated mitochondria, which were also prevented by DKK1 (Fig 8A). Furthermore, it has been described that HKII activity is also required for its protective effect [59]. We used hippocampal slices treated with Wnt3a +/- DKK1 to test whether Wnt signaling also modulates the activity of HK. The activity assay showed increased HK activity in response to Wnt3a, which was inhibited by co-incubation with DKK1, reinforcing our hypothesis that HKII could be a good candidate to mediate the inhibitory effect of Wnt3a on mPTP.

Together, these results suggest that Wnt signaling could regulate inhibition of the mPTP through a mechanism that involves mitochondrial GSK-3β inactivation. It may thereby act by regulating the detachment/translocation process of HKII to mitochondria and/or by GSK-3β binding to the ANT protein to directly inhibit pore opening.

## Discussion

The permeabilization of mitochondrial membranes is determinant for cell fate decision between life or death, and has been described as “a point of no return” in mitochondrial cell death [60]. The mPTP plays a central role in alterations of mitochondrial structure and function leading to neuronal damage related to several neurodegenerative diseases [34,61–63], including AD [7]. This is why the mPTP has been consistently proposed as molecular target for the development of therapeutic approaches in neurodegeneration [64]. In AD the permeability of the mitochondrial membranes has been described as an early event in the pathogenesis of the disease, preceding even the synaptic dysfunction and the neuronal cell death [14,65]. For this reason, we have been interested in determining how to protect the mitochondria, to prevent the toxic events that finally produce the neuronal damage. Our previous studies have shown that activation of the Wnt signaling pathway has a neuroprotective role against Aβ toxicity [21,25]; however, there is no direct evidence that explains whether this neuroprotective effect is mediated by a regulation of cell death cascades that are initiated in the mitochondria. Our data demonstrate that the Wnt3a ligand prevents the Aβo-induced mPTP opening and thereby all of the consequent effects, such as mitochondrial morphological changes, structural alteration, _m_ΔΨ collapse, cytochrome *c* release and, therefore, neuronal cell death. The results obtained in the mPTP opening assay suggest that Wnt signaling could protect neurons from cell death by inhibiting the mPTP induction, as has been described for the classical mPTP inhibitor, CsA [33]. Both, CsA and Wnt3a, provide the same level of mPTP inhibition (Fig 1). Equivalent results were obtained in the ultrastructural analyses and the neuronal viability assay where Wnt-mediated protection reached the same levels as CsA to prevent Aβos damage (Figs 3 and 5C, respectively). The magnitude of the effect of both agents suggests that Wnt signaling prevents neuronal cell death by regulating mPTP opening.

Consistent with the inhibition of the mPTP, we also showed that the morphological changes induced in mitochondria during the permeability transition are prevented by Wnt3a (Fig 2). The electron microscopy analysis on synaptic mitochondria showed dramatic changes in the mitochondrial integrity in slices that were acutely exposed to Aβos only for 1 h. These alterations are correlated with the enhanced sensitivity of synaptic mitochondria to Aβ-induced damage [66] specifically at the CA1 region, where we performed the analysis (S1 Fig) [67]. The early sensitivity of mitochondria to amyloid damage compared with any other compartment in the cell has been extensively discussed by other groups that have proposed the “AD mitochondrial cascade hypothesis”, wherein the common feature of disease progression is early mitochondrial dysfunction [6,14]. Such dysfunction includes the high sensitivity of synaptic mitochondria to permeability transition [68]. Interestingly, at 1 h of Aβos treatment, we did not observe neuronal cell death (S2 Fig), reinforcing the notion that the damage observed in mitochondria is not a consequence of a general neuronal impairment; instead, Aβos only affected mitochondria as an initial step towards cell death, as we observed in the 24-h treatments (Fig 5C).

### How can Wnt signaling regulate the permeability transition and pore inactivation?

Most of the effects that has been described for the Wnt signaling pathway are β-catenin-dependent and/or are mediated by the diversity of Wnt target genes [19]. Likewise, there are several mPTP modulators that have been described to act through interactions with mPTP components that could be related to Wnt signaling and their genes [13,69,70]. One of these modulators is the anti-apoptotic protein Bcl-2 [71], which is downregulated in AD models [72]. We have previously shown that Bcl-2 is a Wnt target gene [73] that participates in mitochondrial stabilization against Aβ damage [74]. In the context of mPTP-induced apoptosis, Bcl-2 has been shown to prevent cell death by interacting with CypD [70], revealing a novel protective function of CypD. However, we observed that the Wnt-mediated mPTP inhibition was not dependent on the activation of Wnt target gene transcription (Fig 6), strengthening the idea that protection is not related to the action of Bcl-2, or to any other Wnt-regulated gene.

It is well-documented that Aβ induces mPTP activation in an AD mouse model and *in vitro* [75,76] by the direct interaction with CypD [77]. In an AD mice model, which presents mitochondrial ultrastructural abnormalities [78] similar to those shown in Fig 2, specifically mitochondrial swelling, CypD deficiency attenuates the cellular death induced by Aβ and improves memory and synaptic function [77]. In the same way, increased levels of CypD in synaptic mitochondria are also important for their sensitivity to Aβ damage, making them more vulnerable to permeability transition processes [79]. These antecedents suggest CypD as the best candidate to control the open conformation of the pore [8], however we did not observe any changes in CypD protein levels (input) in hippocampal slices treated with Wnt3a (Fig 7C). This result does not allow us to discard the participation of CypD in the Wnt-mediated protection, but it enables us to explore other possible candidates. Indeed, CypD has been identified, at least in AD, as a main contributor of neuronal damage through the induction of the mPTP [77,80], however many other proteins have been implicated in the formation of the pore, some of them dependent on CypD, as Bcl-2 [70], and others that are able to form the pore regardless of CypD, such as Bax [12] and ATP synthase [10,81]. Furthermore, glutamate-triggered mPTP has been shown to be produced in both CypD-dependent and -independent manner, according to the concentrations used [82]. This scenario widens the range of candidates that could participate in the Wnt3a-mediated inhibition of the pore.

Recent studies support the idea that GSK-3β, one of the central components of the Wnt signaling pathway, plays a crucial role in the regulation of the mPTP in a variety of disease models [83–85]. Several years ago a mitochondrial GSK-3β pool was detected in cerebellum and since then, GSK-3β has been proposed to participate and regulate the induction of the permeability transition, being a point of convergence of different survival signals [86]. The inactive form of GSK-3β (phosphorylated at Ser9) interacts with ANT, a proposed component of the mPTP [52,56]. This interaction has been correlated with a decrease in the CypD-ANT binding, preventing CypD translocation from the matrix to the mitochondrial inner membrane [87,88], which is a necessary event for Cyp-dependent mPTP formation [77]. Interestingly, the activation of Wnt signaling results in GSK-3β inactivation by its phosphorylation at Ser9, as occurs in mitochondria, producing the inactivation of the cytoplasmic GSK-3β pool, without affecting total GSK-3β levels [24,89]. Our results showed an increase in mitochondrial p-GSK3β-S9 levels in response to Wnt3a (Fig 7A and 7B) and a Wnt-induced interaction between p-GSK3β-S9 and ANT, which suggest that the inhibition of the pore opening, in response to Wnt signaling activation, could be mediated by mitochondrial GSK3β through the regulation of the interaction between the mPTP protein components.

Reinforcing the idea of GSK3β participation in the inhibition of the pore, we showed that inhibiting its kinase activity with the specific inhibitior 6-BIO, which has been widely used as a Wnt signaling activator [90], the Aβos-induced mPTP opening was inhibited at the same magnitude that Wnt3a was in our live cell mPTP-assay (Fig 6B). These results support the antecedents related to the role of p-GSK3β-S9 in the inhibition of the pore, and strengthens our hypothesis of its participation in the protective effect of Wnt3a.

**Fig 9:**
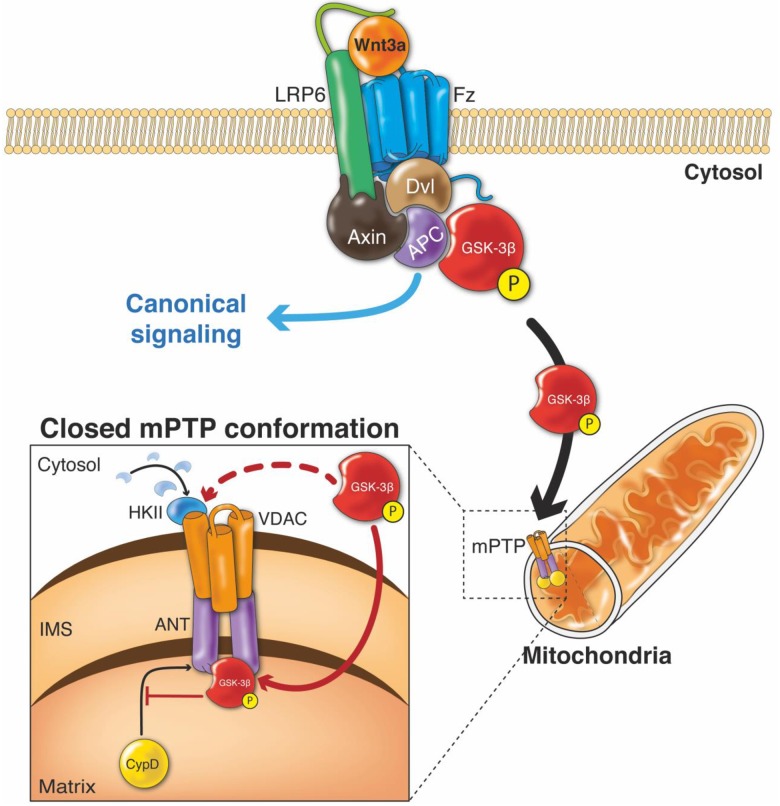
Proposed mechanism for the action of Wnt3a in regulating mitochondrial mPTP opening. Wnt signaling is activated by binding of the Wnt3a ligand to its receptor, Fz, and to its co-receptor, LRP6. This interaction activates Dvl, which causes the dissociation of the destruction complex, which includes Axin, APC, and GSK-3β. This dissociation prevents the proteasomal degradation of β-catenin, thereby inducing its cytoplasmic accumulation and eventual translocation to the nucleus, where it regulates the expression of Wnt target genes. This pathway is known as Wnt/β-catenin signaling. However, Wnt can also act through another signaling mediator, GSK-3β, which has functions that are independent of β-catenin and thus of Wnt target gene transcription [94]. The activation of Wnt signaling triggers the inhibitory phosphorylation of GSK-3β at serine 9 in the cytosol. In our proposed model, the inhibited GSK-3β accumulates in mitochondria in response to the activation of Wnt signaling by Wnt3a. Mitochondrial p-GSK3β-S9 then interacts with ANT to inhibit the opening of the mPTP. This interaction has also been correlated with inhibition of the binding between ANT and CypD that is necessary for the conformational shift and opening of the mPTP. GSK-3β activity can also control the detachment or translocation of HKII to the mitochondria. Activation of Wnt signaling increases mitochondrial HKII, probably through the inhibition of GSK-3β activity, thus also favoring the closed conformation of the pore. (Fz: Frizzled; Dvl: Dishevelled; APC: adenomatous polyposis coli; GSK-3β: glycogen synthase kinase-3β; p-GSK3β-S9: GSK-3β phosphorylated at Ser9; ANT: adenine nucleotide translocase; CypD: cyclophilin D; VDAC: voltage-dependent anion channel; HKII: hexokinase II).

Another known mPTP modulator is the mitochondrial hexokinase II (HKII). Detachment of HKII from the mitochondria induces mPTP opening and cell death [57,91]. Interestingly, and consistent with the proposed role of p-GSK3β-S9 in mPTP inhibition, it has been described that the inactivating phosphorylation of GSK-3β by Akt favors the association of HKII with the outer mitochondrial membrane [92]. By contrast, activation of GSK-3β has been shown to induce the release of HKII, increasing susceptibility to cell death [13,93]. Here, we observed increased levels of HKII on the mitochondria in response to Wnt3a (Fig 8A). Moreover, not only mitochondrial binding of HKII is required to inhibit mPTP. As has been shown, HKII activity also contributes to the protective effect of the kinase [59]. We explained that Wnt3a increases HK activity in hippocampal slices, possibly favoring the protective effect of Wnt3a on mPTP inhibition. These results suggest another possible mechanism for Wnt/GSK-3β signaling in the regulation of mPTP opening, and it allows us to propose two distinct but complementary modes of regulation: (1) via phosphorylation of the cytoplasmic pool of GSK-3β and thereby the translocation and attachment of HKII to the mitochondria to inhibit mPTP opening and (2) via regulation of mitochondrial p-GSK3β-S9 levels to directly interact with the ANT protein in the mPTP complex (Fig 9).

We proposed, for the first time, a possible mechanism to explain the neuroprotective action of Wnt signaling, which involves the regulation of the mPTP through the inhibition of GSK-3β, thereby preventing the Aβos neurotoxicity. This study suggests a possible new approach for the treatment of AD, based on the preservation of the mitochondrial structure as a target, and opens a new line of study in the field of Wnt signaling in neuroprotection.

## Supporting Information

S1 Dataset Time-lapse images (mitochondrial calcein in FITC channel) of a control neuron treated with Aβos at 300s. Images were used to prepare Fig 1 panels and for the analysis.
(AVI)

S2 Dataset Time-lapse images (mitochondrial calcein in FITC channel) of a Wnt3a-treated neuron and exposed to Aβos at 300s. Images were used to prepare Fig 1 panels and for the analysis.
(AVI)

S3 Dataset Raw data obtained from PTP assays and used to generate Figs 1 and 6 graphs. (PZF)

S4 Dataset Raw data obtained from PTP assays in response to calcium and used to generate Fig 2 graph. (PZF)

S5 Dataset Raw data obtained from the analysis of electron microscopy images and used to generate Fig 3 graphs. (PZF)

S6 Dataset Raw data of the 3D reconstruction analysis and used to generate Fig 4 graphs. (PZF)

S7 Dataset Raw data used to generate Fig 5 graph for the live-dead assay. (PZF)

S8 Dataset Raw data used to generate Fig 5 graph for the cytochrome c release assay. (PZF)

S9 Dataset Raw data used to generate Figs 5 and 6 graphs, corresponding to the mitochondrial membrane potential assays. (PZF)

S10 Dataset Raw data used to generate Fig 7 graph for the mitochondrial p-GSK3β levels from Western blots densitometry. (PZF)

S11 Dataset Raw data used to generate Fig 7 graph, obtained from the densitometric analyses of Western blots and immunoprecipitation assays. (PZFX)

S12 Dataset Raw data used to generate Fig 8 graph for the hexokinase activity assay. (PZF)

S1 Fig Mitochondrial morphology analysis by electron microscopy. **(A)** Representative image of a hippocampal slice stained with toluidine blue (scale bar, 1mm). The black square shows the CA1 region selected for the analysis. A close-up from the image is shown in the right panel (scale bar, 100 μm). (B) Representative images of different treatments. Images were acquired with an electron microscope without digital magnification (16,500X). Mitochondria were pseudocolored (orange) to differentiate them from other structures. Scale bars, 1 μm. (C) Quantification of the number of mitochondria per area from electron microscopy images. Hundred μm2 area correspond to the whole area of the image obtained at 16.500 X.
(TIF)

S2 Fig Neuronal viability is not affected in hippocampal slices after 1h Aβo-exposure. Mouse hippocampal slices (400 μm) were pre-incubated for 4h with Wnt3a and then treated with 5μM Aβo for 1 h. Slices were fixed and processed for Hoechst staining. Images show a representative hippocampal slice stained with Hoechst (a-d). Graph shows the quantification of percentage of apoptotic nuclei in each condition (e). Non-significant changes were observed between each condition using one-way ANOVA test with a *post hoc* Bonferroni. Quantifications represent the results of three independent experiments.
(TIFF)

S3 Fig Wnt3a prevents apoptosis induced by Aβo in hippocampal neurons. Neurons were co-incubated with Wnt3a protein and 5μM Aβo for 24 h. Apoptotic nuclei were detected with Hoechst stain (1μg/ml) in fixed neurons (a-d). Magnification shows representative nucleus of neurons treated with control media (a’), Aβo (b’), Wnt3a+Aβo (c’) and Wnt3a alone (d’). Graph shows the quantification of percentage of apoptotic nuclei in each condition (e). Statistical analysis in both experiments was carried out using one-way ANOVA test with a *post hoc* Bonferroni with ***p<0,0005. Quantifications represent the results of six independent experiments.
(TIFF)

S1 File Supplementary Materials and Methods. (DOCX)

S2 File Supplementary raw data file containing the original and scanned blots use to prepare figure panels of Figs 7 and 8. (DOC)

## References

[1] Hardy J, Selkoe DJ. The amyloid hypothesis of Alzheimer’s disease: progress and problems on the road to therapeutics. Science. 2002;297: 353–356. 10.1126/science.1072994 12130773

[2] Walsh DM, Klyubin I, Fadeeva J V, Cullen WK, Anwyl R, Wolfe MS, et al Naturally secreted oligomers of amyloid beta protein potently inhibit hippocampal long-term potentiation in vivo. Nature. 2002;416: 535–9. 10.1038/416535a 11932745

[3] Li S, Hong S, Shepardson NE, Walsh DM, Shankar GM, Selkoe D. Soluble oligomers of amyloid beta protein facilitate hippocampal long-term depression by disrupting neuronal glutamate uptake. Neuron. Elsevier Ltd; 2009;62: 788–801. 10.1016/j.neuron.2009.05.012 PMC2702854 19555648

[4] Ferreira ST, Klein WL. The Aβ oligomer hypothesis for synapse failure and memory loss in Alzheimer’s disease. Neurobiol Learn Mem. 2011;96: 529–43. 10.1016/j.nlm.2011.08.003 21914486 PMC4390395

[5] Supnet C, Bezprozvanny I. Neuronal calcium signaling, mitochondrial dysfunction, and Alzheimer’s disease. J Alzheimers Dis. 2010;20 Suppl 2: S487–98. 20413848 10.3233/JAD-2010-100306 PMC4996661

[6] Swerdlow RH, Burns JM, Khan SM. The Alzheimer’s disease mitochondrial cascade hypothesis. J Alzheimers Dis. 2010;20 Suppl 2: S265–79. 20442494 10.3233/JAD-2010-100339 PMC2883665

[7] Du H, Yan SS. Mitochondrial permeability transition pore in Alzheimer’s disease: cyclophilin D and amyloid beta. Biochim Biophys Acta. Elsevier B.V.; 2010;1802: 198–204. 10.1016/j.bbadis.2009.07.005 PMC3280723 19616093

[8] Schinzel AC, Takeuchi O, Huang Z, Fisher JK, Zhou Z, Rubens J, et al Cyclophilin D is a component of mitochondrial permeability transition and mediates neuronal cell death after focal cerebral ischemia. Proc Natl Acad Sci U S A. 2005;102: 12005–10. 10.1073/pnas.0505294102 16103352 PMC1189333

[9] Baines CP, Kaiser RA, Purcell NH, Blair NS, Osinska H, Hambleton MA, et al Loss of cyclophilin D reveals a critical role for mitochondrial permeability transition in cell death. Nature. 2005;434: 658–62. 10.1038/nature03434 15800627

[10] Bernardi P, Rasola A, Forte M, Lippe G. The Mitochondrial Permeability Transition Pore: Channel Formation by F-ATP Synthase, Integration in Signal Transduction, and Role in Pathophysiology. Physiol Rev. American Physiological Society; 2015;95: 1111–55. 10.1152/physrev.00001.2015 PMC4600949 26269524

[11] Carraro M, Giorgio V, Šileikytė J, Sartori G, Forte M, Lippe G, et al Channel formation by yeast F-ATP synthase and the role of dimerization in the mitochondrial permeability transition. J Biol Chem. American Society for Biochemistry and Molecular Biology; 2014;289: 15980–5. 10.1074/jbc.C114.559633 PMC4047373 24790105

[12] Li T, Brustovetsky T, Antonsson B, Brustovetsky N. Oligomeric BAX induces mitochondrial permeability transition and complete cytochrome c release without oxidative stress. Biochim Biophys Acta. NIH Public Access; 2008;1777: 1409–21. 10.1016/j.bbabio.2008.08.002 PMC2613194 18771651

[13] Arrázola MS, Silva-Alvarez C, Inestrosa NC. How the Wnt signaling pathway protects from neurodegeneration: the mitochondrial scenario. Front Cell Neurosci. 2015;9: 1–13. 25999816 10.3389/fncel.2015.00166 PMC4419851

[14] Pagani L, Eckert A. Amyloid-Beta interaction with mitochondria. Int J Alzheimers Dis. 2011;2011: 925050 10.4061/2011/925050 21461357 PMC3065051

[15] Orellana AMM, Vasconcelos AR, Leite JA, de Sá Lima L, Andreotti DZ, Munhoz CD, et al Age-related neuroinflammation and changes in AKT-GSK-3β and WNT/ β-CATENIN signaling in rat hippocampus. Aging (Albany NY). Impact Journals, LLC; 2015;7: 1094–111. 10.18632/aging.100853 PMC4712335 26647069

[16] De Ferrari G V, Inestrosa NC. Wnt signaling function in Alzheimer’s disease. Brain Res Brain Res Rev. 2000;33: 1–12. 10967351 10.1016/s0165-0173(00)00021-7

[17] De Ferrari G V, Chacón M a, Barría MI, Garrido JL, Godoy J a, Olivares G, et al Activation of Wnt signaling rescues neurodegeneration and behavioral impairments induced by beta-amyloid fibrils. Mol Psychiatry. 2003;8: 195–208. 10.1038/sj.mp.4001208 12610652

[18] Liu C-C, Tsai C-W, Deak F, Rogers J, Penuliar M, Sung YM, et al Deficiency in LRP6-Mediated Wnt Signaling Contributes to Synaptic Abnormalities and Amyloid Pathology in Alzheimer’s Disease. Neuron. Elsevier Inc.; 2014;84: 63–77. 10.1016/j.neuron.2014.08.048 PMC4199382 25242217

[19] Clevers H, Nusse R. Wnt/β-catenin signaling and disease. Cell. 2012;149: 1192–205. 10.1016/j.cell.2012.05.012 22682243

[20] Arrázola MS, Varela-Nallar L, Colombres M, Toledo EM, Cruzat F, Pavez L, et al Calcium/calmodulin-dependent protein kinase type IV is a target gene of the Wnt/beta-catenin signaling pathway. J Cell Physiol. 2009;221: 658–67. 10.1002/jcp.21902 19711354

[21] Cerpa W, Toledo EM, Varela-Nallar L, Inestrosa NC. The role of Wnt signaling in neuroprotection. Drug News Perspect. 2009;22: 579–91. 10.1358/dnp.2009.10.1436817 20140278

[22] De Ferrari G V, Avila ME, Medina MA, Perez-Palma E, Bustos BI, Alarcon MA. Wnt/β-catenin signaling in Alzheimer’s disease. CNS Neurol Disord Drug Targets. 2014;13: 745–54. 24365184 10.2174/1871527312666131223113900

[23] Toledo EM, Inestrosa NC. Wnt signaling activation reduces neuropathological markers in a mouse model of Alzheimer’s disease. Mol Psychiatry. Nature Publishing Group; 2010;15: 228–228. 10.1038/mp.2009.72 19621015

[24] Alvarez AR, Godoy J a, Mullendorff K, Olivares GH, Bronfman M, Inestrosa NC. Wnt-3a overcomes beta-amyloid toxicity in rat hippocampal neurons. Exp Cell Res. 2004;297: 186–96. 10.1016/j.yexcr.2004.02.028 15194435

[25] Inestrosa NC, Varela-Nallar L. Wnt signaling in the nervous system and in Alzheimer’s disease. J Mol Cell Biol. 2014;6: 64–74. 10.1093/jmcb/mjt051 24549157

[26] Toledo EM, Inestrosa NC. Activation of Wnt signaling by lithium and rosiglitazone reduced spatial memory impairment and neurodegeneration in brains of an APPswe/PSEN1DeltaE9 mouse model of Alzheimer’s disease. Mol Psychiatry. Nature Publishing Group; 2010;15: 272–85, 228. 10.1038/mp.2009.72 19621015

[27] Vargas JY, Fuenzalida M, Inestrosa NC. In vivo Activation of Wnt Signaling Pathway Enhances Cognitive Function of Adult Mice and Reverses Cognitive Deficits in an Alzheimer’s Disease Model. J Neurosci. 2014;34: 2191–202. 10.1523/JNEUROSCI.0862-13.2014 24501359 PMC6608527

[28] Klein WL. Abeta toxicity in Alzheimer’s disease: globular oligomers (ADDLs) as new vaccine and drug targets. Neurochem Int. 2002;41: 345–52. 12176077 10.1016/s0197-0186(02)00050-5

[29] Godoy JA, Allard C, Arrázola MS, Zolezzi JM, Inestrosa NC. SIRT1 Protects Dendrites, Mitochondria and Synapses from Aβ Oligomers in Hippocampal Neurons. J Alzheimers Dis Park. 2013;3.

[30] Silva-Alvarez C, Arrázola MS, Godoy J a, Ordenes D, Inestrosa NC. Canonical Wnt signaling protects hippocampal neurons from Aβ oligomers: role of non-canonical Wnt-5a/Ca(2+) in mitochondrial dynamics. Front Cell Neurosci. 2013;7: 97 10.3389/fncel.2013.00097 23805073 PMC3691552

[31] Gillessen T, Grasshoff C, Szinicz L. Mitochondrial permeability transition can be directly monitored in living neurons. Biomed Pharmacother. 2002;56: 186–93. 12109811 10.1016/s0753-3322(02)00184-1

[32] Arrázola MS, Inestrosa NC. Monitoring mitochondrial membranes permeability in live neurons and mitochondrial swelling through electron microscopy analysis In: Lossi L, Merighi A, editors. Neuronal Cell Death Methods in Molecular Biology. Springer New York; 2015 pp. 1254, 87–97. 10.1007/978-1-4939-2152-2_7 25431059

[33] Halestrap AP, Connern CP, Griffiths EJ, Kerr PM. Cyclosporin A binding to mitochondrial cyclophilin inhibits the permeability transition pore and protects hearts from ischaemia/reperfusion injury. Mol Cell Biochem. 1997;174: 167–72. 9309682

[34] Barrientos SA, Martinez NW, Yoo S, Jara JS, Zamorano S, Hetz C, et al Axonal degeneration is mediated by the mitochondrial permeability transition pore. J Neurosci. 2011;31: 966–78. 10.1523/JNEUROSCI.4065-10.2011 21248121 PMC3245862

[35] Korkotian E, Segal M. Calcium-containing organelles display unique reactivity to chemical stimulation in cultured hippocampal neurons. J Neurosci. 1997;17: 1670–82. 9030626 10.1523/JNEUROSCI.17-05-01670.1997 PMC6573380

[36] Song DD, Shults CW, Sisk A, Rockenstein E, Masliah E. Enhanced substantia nigra mitochondrial pathology in human alpha-synuclein transgenic mice after treatment with MPTP. Exp Neurol. 2004;186: 158–72. 10.1016/S0014-4886(03)00342-X 15026254

[37] Picard M, White K, Turnbull DM. Mitochondrial morphology, topology, and membrane interactions in skeletal muscle: a quantitative three-dimensional electron microscopy study. J Appl Physiol. 2013;114: 161–71. 10.1152/japplphysiol.01096.2012 23104694 PMC3544498

[38] Sun MG, Williams J, Munoz-Pinedo C, Perkins G a, Brown JM, Ellisman MH, et al Correlated three-dimensional light and electron microscopy reveals transformation of mitochondria during apoptosis. Nat Cell Biol. 2007;9: 1057–65. 10.1038/ncb1630 17721514

[39] Godoy JA, Arrázola MS, Ordenes D, Silva-Alvarez C, Braidy N, Inestrosa NC. Wnt-5a ligand modulates mitochondrial fission-fusion in rat hippocampal neurons. J Biol Chem. 2014;289: 36179–93. 10.1074/jbc.M114.557009 25336659 PMC4276881

[40] Marchionni I, Kasap Z, Mozrzymas JW, Sieghart W, Cherubini E, Zacchi P. New insights on the role of gephyrin in regulating both phasic and tonic GABAergic inhibition in rat hippocampal neurons in culture. Neuroscience. 2009;164: 552–62. 10.1016/j.neuroscience.2009.07.063 19660531

[41] Brustovetsky T, Li V, Brustovetsky N. Stimulation of glutamate receptors in cultured hippocampal neurons causes Ca2+-dependent mitochondrial contraction. Cell Calcium. 2009;46: 18–29. 10.1016/j.ceca.2009.03.017 19409612 PMC2703686

[42] Buckman JF, Hernández H, Kress GJ, Votyakova T V, Pal S, Reynolds IJ. MitoTracker labeling in primary neuronal and astrocytic cultures: influence of mitochondrial membrane potential and oxidants. J Neurosci Methods. 2001;104: 165–76. 11164242 10.1016/s0165-0270(00)00340-x

[43] Saraiva LM, Seixas da Silva GS, Galina A, Da-Silva WS, Klein WL, Ferreira ST, et al Amyloid-β triggers the release of neuronal hexokinase 1 from mitochondria. PLoS One. 2010;5: e15230 10.1371/journal.pone.0015230 21179577 PMC3002973

[44] Bertoni JM. Competitive Inhibition of Rat Brain Hexokinase by 2-Deoxyglucose, Glucosamine, and Metrizamide. J Neurochem. Blackwell Publishing Ltd; 1981;37: 1523–1528. 10.1111/j.1471-4159.1981.tb06322.x 7334375

[45] Tsai CS, Chen Q. Purification and kinetic characterization of hexokinase and glucose-6-phosphate dehydrogenase from Schizosaccharomyces pombe. Biochem Cell Biol. 1998;76: 107–13. 9666312 10.1139/o98-001

[46] Kawamoto EM, Gleichmann M, Yshii LM, Lima L de S, Mattson MP, Scavone C. Effect of activation of canonical Wnt signaling by the Wnt-3a protein on the susceptibility of PC12 cells to oxidative and apoptotic insults. Braz J Med Biol Res. Associação Brasileira de Divulgação Científica; 2012;45: 58–67. 10.1590/S0100-879X2011007500157 PMC3854138 22124704

[47] Petronilli V, Miotto G, Canton M, Brini M, Colonna R, Bernardi P, et al Transient and long-lasting openings of the mitochondrial permeability transition pore can be monitored directly in intact cells by changes in mitochondrial calcein fluorescence. Biophys J. 1999;76: 725–34. 10.1016/S0006-3495(99)77239-5 9929477 PMC1300077

[48] Kawano Y, Kypta R. Secreted antagonists of the Wnt signalling pathway. J Cell Sci. 2003;116: 2627–34. 10.1242/jcs.00623 12775774

[49] Moreira PI, Santos MS, Moreno A, Rego AC, Oliveira C. Effect of amyloid beta-peptide on permeability transition pore: a comparative study. J Neurosci Res. 2002;69: 257–67. 10.1002/jnr.10282 12111807

[50] Emami KH, Nguyen C, Ma H, Kim DH, Jeong KW, Eguchi M, et al A small molecule inhibitor of beta-catenin/CREB-binding protein transcription. Proc Natl Acad Sci U S A. 2004;101: 12682–7. 10.1073/pnas.0404875101 15314234 PMC515116

[51] Tanno M, Kuno A, Ishikawa S, Miki T, Kouzu H, Yano T, et al Translocation of Glycogen Synthase Kinase-3β (GSK-3β), a Trigger of Permeability Transition, Is Kinase Activity-dependent and Mediated by Interaction with Voltage-dependent Anion Channel 2 (VDAC2). J Biol Chem. 2014;289: 29285–96. 10.1074/jbc.M114.563924 25187518 PMC4200279

[52] Nishihara M, Miura T, Miki T, Tanno M, Yano T, Naitoh K, et al Modulation of the mitochondrial permeability transition pore complex in GSK-3beta-mediated myocardial protection. J Mol Cell Cardiol. 2007;43: 564–70. 10.1016/j.yjmcc.2007.08.010 17931653

[53] Fu H, Xu H, Chen H, Li Y, Li W, Zhu Q, et al Inhibition of glycogen synthase kinase 3 ameliorates liver ischemia/reperfusion injury via an energy-dependent mitochondrial mechanism. J Hepatol. 2014;61: 816–24. 10.1016/j.jhep.2014.05.017 24862449

[54] Wang Z, Ge Y, Bao H, Dworkin L, Peng A, Gong R. Redox-sensitive glycogen synthase kinase 3β-directed control of mitochondrial permeability transition: rheostatic regulation of acute kidney injury. Free Radic Biol Med. 2013;65: 849–58. 10.1016/j.freeradbiomed.2013.08.169 23973862 PMC3859848

[55] Stambolic V, Woodgett JR. Mitogen inactivation of glycogen synthase kinase-3 beta in intact cells via serine 9 phosphorylation. Biochem J. 1994;303 (Pt 3: 701–4. 7980435 10.1042/bj3030701 PMC1137602

[56] Juhaszova M, Zorov DB, Kim S, Pepe S, Fu Q, Fishbein KW, et al Glycogen synthase kinase-3beta mediates convergence of protection signaling to inhibit the mitochondrial permeability transition pore. J Clin Invest. 2004;113: 1535–1549. 10.1172/JCI19906 15173880 PMC419483

[57] Rasola A, Sciacovelli M, Pantic B, Bernardi P. Signal transduction to the permeability transition pore. FEBS Lett. Federation of European Biochemical Societies; 2010;584: 1989–96. 10.1016/j.febslet.2010.02.022 PMC2866765 20153328

[58] Reddy PH. Amyloid beta-induced glycogen synthase kinase 3β phosphorylated VDAC1 in Alzheimer’s disease: Implications for synaptic dysfunction and neuronal damage. Biochim Biophys Acta. Elsevier B.V.; 2013;1832: 1913–1921. 10.1016/j.bbadis.2013.06.012 PMC3825775 23816568

[59] Sun L, Shukair S, Naik TJ, Moazed F, Ardehali H. Glucose phosphorylation and mitochondrial binding are required for the protective effects of hexokinases I and II. Mol Cell Biol. American Society for Microbiology (ASM); 2008;28: 1007–17. 10.1128/MCB.00224-07 PMC2223386 18039843

[60] Galluzzi L, Blomgren K, Kroemer G. Mitochondrial membrane permeabilization in neuronal injury. Nat Rev Neurosci. Nature Publishing Group; 2009;10: 481–94. 10.1038/nrn2665 19543220

[61] Warne J, Pryce G, Hill J, Shi X, Lennerås F, Puentes F, et al Selective inhibition of the mitochondrial permeability transition pore protects against neuro-degeneration in experimental multiple sclerosis. J Biol Chem. 2015; 10.1074/jbc.M115.700385 PMC4813465 26679998

[62] Quintanilla RA, Jin YN, von Bernhardi R, Johnson GVW. Mitochondrial permeability transition pore induces mitochondria injury in Huntington disease. Mol Neurodegener. 2013;8: 45 10.1186/1750-1326-8-45 24330821 PMC3878840

[63] Martin LJ, Semenkow S, Hanaford A, Wong M. Mitochondrial permeability transition pore regulates Parkinson’s disease development in mutant α-synuclein transgenic mice. Neurobiol Aging. 2014;35: 1132–52. 10.1016/j.neurobiolaging.2013.11.008 24325796 PMC3948207

[64] Rao VK, Carlson E a, Yan SS. Mitochondrial permeability transition pore is a potential drug target for neurodegeneration. Biochim Biophys Acta. Elsevier B.V.; 2014;1842: 1267–1272. 10.1016/j.bbadis.2013.09.003 PMC3991756 24055979

[65] Du H, Guo L, Yan S, Sosunov A a, McKhann GM, Yan SS. Early deficits in synaptic mitochondria in an Alzheimer’s disease mouse model. Proc Natl Acad Sci U S A. 2010;107: 18670–5. 10.1073/pnas.1006586107 20937894 PMC2972922

[66] Du H, Guo L, Yan SS. Synaptic mitochondrial pathology in Alzheimer’s disease. Antioxid Redox Signal. 2012;16: 1467–75. 10.1089/ars.2011.4277 21942330 PMC3329948

[67] Balietti M, Giorgetti B, Casoli T, Solazzi M, Tamagnini F, Burattini C, et al Early selective vulnerability of synapses and synaptic mitochondria in the hippocampal CA1 region of the Tg2576 mouse model of Alzheimer’s disease. J Alzheimers Dis. 2013;34: 887–96. 10.3233/JAD-121711 23313923

[68] Brown MR, Sullivan PG, Geddes JW. Synaptic mitochondria are more susceptible to Ca2+overload than nonsynaptic mitochondria. J Biol Chem. 2006;281: 11658–68. 10.1074/jbc.M510303200 16517608

[69] Rasola A, Sciacovelli M, Chiara F, Pantic B, Brusilow WS, Bernardi P. Activation of mitochondrial ERK protects cancer cells from death through inhibition of the permeability transition. Proc Natl Acad Sci U S A. 2010;107: 726–31. 10.1073/pnas.0912742107 20080742 PMC2818893

[70] Eliseev R a, Malecki J, Lester T, Zhang Y, Humphrey J, Gunter TE. Cyclophilin D interacts with Bcl2 and exerts an anti-apoptotic effect. J Biol Chem. 2009;284: 9692–9. 10.1074/jbc.M808750200 19228691 PMC2665090

[71] Scorrano L, Korsmeyer SJ. Mechanisms of cytochrome c release by proapoptotic BCL-2 family members. Biochem Biophys Res Commun. 2003;304: 437–444. 12729577 10.1016/s0006-291x(03)00615-6

[72] Paradis E, Koutroumanis M, Goodyer C. Amyloid Beta Peptide of Alzheimer ‘ s Disease Downregulates Bcl-2 and Upregulates Bax Expression in Human Neurons. 1996;16: 7533–7539. 10.1523/JNEUROSCI.16-23-07533.1996 PMC6579094 8922409

[73] Fuentealba RA, Farias G, Scheu J, Bronfman M, Marzolo MP, Inestrosa NC. Signal transduction during amyloid-beta-peptide neurotoxicity: role in Alzheimer disease. Brain Res Brain Res Rev. 2004;47: 275–89. 10.1016/j.brainresrev.2004.07.018 15572177

[74] Fuenzalida K, Quintanilla R, Ramos P, Piderit D, Fuentealba R a, Martinez G, et al Peroxisome proliferator-activated receptor gamma up-regulates the Bcl-2 anti-apoptotic protein in neurons and induces mitochondrial stabilization and protection against oxidative stress and apoptosis. J Biol Chem. 2007;282: 37006–15. 10.1074/jbc.M700447200 17965419

[75] Shevtzova EF, Kireeva EG, Bachurin SO. Effect of beta-amyloid peptide fragment 25–35 on nonselective permeability of mitochondria. Bull Exp Biol Med. 2001;132: 1173–6. 12152879 10.1023/a:1014559331402

[76] Moreira PI, Santos MS, Moreno A, Oliveira C. Amyloid beta-peptide promotes permeability transition pore in brain mitochondria. Biosci Rep. 2001;21: 789–800. 12166828 10.1023/a:1015536808304

[77] Du H, Guo L, Fang F, Chen D, Sosunov A a, McKhann GM, et al Cyclophilin D deficiency attenuates mitochondrial and neuronal perturbation and ameliorates learning and memory in Alzheimer’s disease. Nat Med. 2008;14: 1097–105. 10.1038/nm.1868 18806802 PMC2789841

[78] Kim MJ, Huh YH, Choi KJ, Jun S, Je a R, Chae H, et al Ultrastructural Abnormalities in APP/PSEN1 Transgenic Mouse Brain as the Alzheimer’s Disease Model. Korean J Microsc. 2012;42: 179–185.

[79] Naga KK, Sullivan PG, Geddes JW. High cyclophilin D content of synaptic mitochondria results in increased vulnerability to permeability transition. J Neurosci. 2007;27: 7469–75. 10.1523/JNEUROSCI.0646-07.2007 17626207 PMC6672616

[80] Guo L, Du H, Yan S, Wu X, McKhann GM, Chen JX, et al Cyclophilin D deficiency rescues axonal mitochondrial transport in Alzheimer’s neurons. PLoS One. 2013;8: e54914 10.1371/journal.pone.0054914 23382999 PMC3561411

[81] Giorgio V, von Stockum S, Antoniel M, Fabbro A, Fogolari F, Forte M, et al Dimers of mitochondrial ATP synthase form the permeability transition pore. Proc Natl Acad Sci U S A. National Academy of Sciences; 2013;110: 5887–92. 10.1073/pnas.1217823110 PMC3625323 23530243

[82] Li V, Brustovetsky T, Brustovetsky N. Role of cyclophilin D-dependent mitochondrial permeability transition in glutamate-induced calcium deregulation and excitotoxic neuronal death. Exp Neurol. NIH Public Access; 2009;218: 171–82. 10.1016/j.expneurol.2009.02.007 PMC2710407 19236863

[83] Hernández-Reséndiz S, Zazueta C. PHO-ERK1/2 interaction with mitochondria regulates the permeability transition pore in cardioprotective signaling. Life Sci. 2014;108: 13–21. 10.1016/j.lfs.2014.04.037 24835217

[84] Rana A, Sharma S. Mechanism of Sphingosine-1-Phosphate induced cardioprotection against I/R injury in diabetic rat heart: Possible involvement of Glycogen synthase kinase 3β and mitochondrial permeability transition pore. Clin Exp Pharmacol Physiol. 2015; 10.1111/1440-1681.12516 26582369

[85] Linkermann A, Konstantinidis K, Kitsis RN. Catch me if you can: targeting the mitochondrial permeability transition pore in myocardial infarction. Cell Death Differ. Nature Publishing Group; 2016;23: 1–2. 10.1038/cdd.2015.151 PMC4815971 26586571

[86] Chiara F, Rasola A. GSK-3 and mitochondria in cancer cells. Front Oncol. 2013;3: 16 10.3389/fonc.2013.00016 23386998 PMC3564062

[87] Miura T, Nishihara M, Miki T. Drug development targeting the glycogen synthase kinase-3beta (GSK-3beta)-mediated signal transduction pathway: role of GSK-3beta in myocardial protection against ischemia/reperfusion injury. J Pharmacol Sci. 2009;109: 162–7. 19179805 10.1254/jphs.08r27fm

[88] Zorov DB, Juhaszova M, Yaniv Y, Nuss HB, Wang S, Sollott SJ. Regulation and pharmacology of the mitochondrial permeability transition pore. Cardiovasc Res. 2009;83: 213–25. 10.1093/cvr/cvp151 19447775 PMC2701724

[89] Nusse R. Wnt signaling. Cold Spring Harb Perspect Biol. 2012;4: 1–3. 10.1101/cshperspect.a011163 PMC3331700 22550232

[90] Tapia‑Rojas C, Schüller A, Lindsay CB, Ureta RC, Mejías‑Reyes C, Hancke J, et al Andrographolide activates the canonical Wnt signalling pathway by a mechanism that implicates the non-ATP competitive inhibition of GSK-3β: autoregulation of GSK-3β in vivo. Biochem J. 2015;466: 415–430. 10.1042/BJ20140207 25423492

[91] Chiara F, Castellaro D, Marin O, Petronilli V, Brusilow WS, Juhaszova M, et al Hexokinase II detachment from mitochondria triggers apoptosis through the permeability transition pore independent of voltage-dependent anion channels. PLoS One. 2008;3: e1852 10.1371/journal.pone.0001852 18350175 PMC2267038

[92] Miyamoto S, Murphy AN, Brown JH. Akt mediates mitochondrial protection in cardiomyocytes through phosphorylation of mitochondrial hexokinase-II. Cell Death Differ. 2008;15: 521–9. 10.1038/sj.cdd.4402285 18064042

[93] Robey RB, Hay N. Mitochondrial hexokinases, novel mediators of the antiapoptotic effects of growth factors and Akt. Oncogene. 2006;25: 4683–96. 10.1038/sj.onc.1209595 16892082

[94] Wu D, Pan W. GSK3: a multifaceted kinase in Wnt signaling. Trends Biochem Sci. 2010;35: 161–8. 10.1016/j.tibs.2009.10.002 19884009 PMC2834833

