# Supplementary figures and images for: Wnt Signaling Prevents the Aβ Oligomer-Induced Mitochondrial Permeability Transition Pore Opening Preserving Mitochondrial Structure in Hippocampal Neurons
Source: PLoS One. 2017 Jan 6;12(1):e0168840. doi: 10.1371/journal.pone.0168840 (PMC5218554; doi:10.1371/journal.pone.0168840)

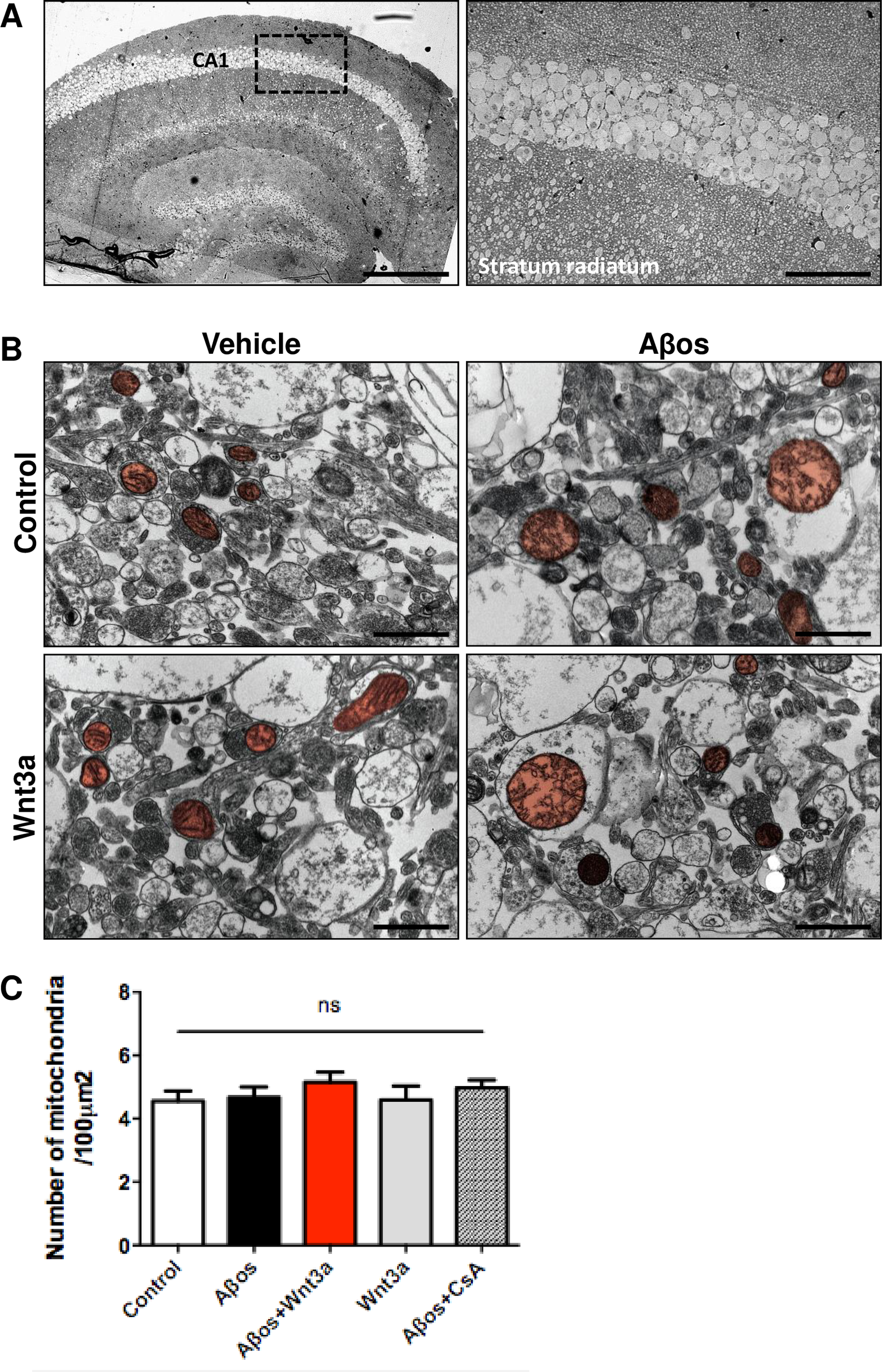

Supplement: S1 Fig — (A) Representative image of a hippocampal slice stained with toluidine blue (scale bar, 1mm). The black square shows the CA1 region selected for the analysis. A close-up from the image is shown in the right panel (scale bar, 100 μm). (B) Representative images of different treatments. Images were acquired with an electron microscope without digital magnification (16,500X). Mitochondria were pseudocolored (orange) to differentiate them from other structures. Scale bars, 1 μm. (C) Quantification of the number of mitochondria per area from electron microscopy images. Hundred μm2 area correspond to the whole area of the image obtained at 16.500 X. (TIF) [file pone.0168840.s013.tif]

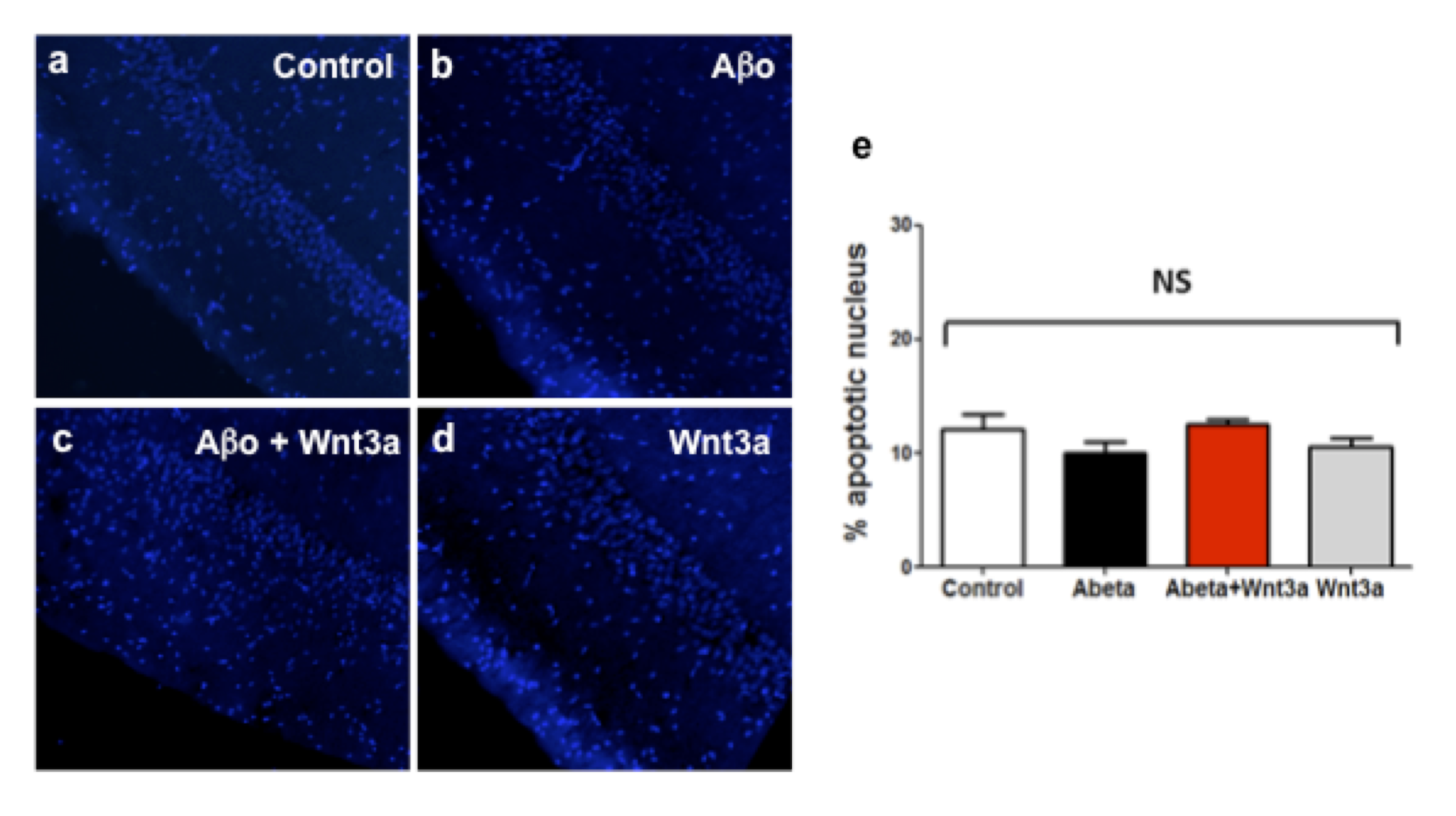

Supplement: S2 Fig — Mouse hippocampal slices (400 μm) were pre-incubated for 4h with Wnt3a and then treated with 5μM Aβo for 1 h. Slices were fixed and processed for Hoechst staining. Images show a representative hippocampal slice stained with Hoechst (a-d). Graph shows the quantification of percentage of apoptotic nuclei in each condition (e). Non-significant changes were observed between each condition using one-way ANOVA test with a post hoc Bonferroni. Quantifications represent the results of three independent experiments. (TIFF) [file pone.0168840.s014.tiff]

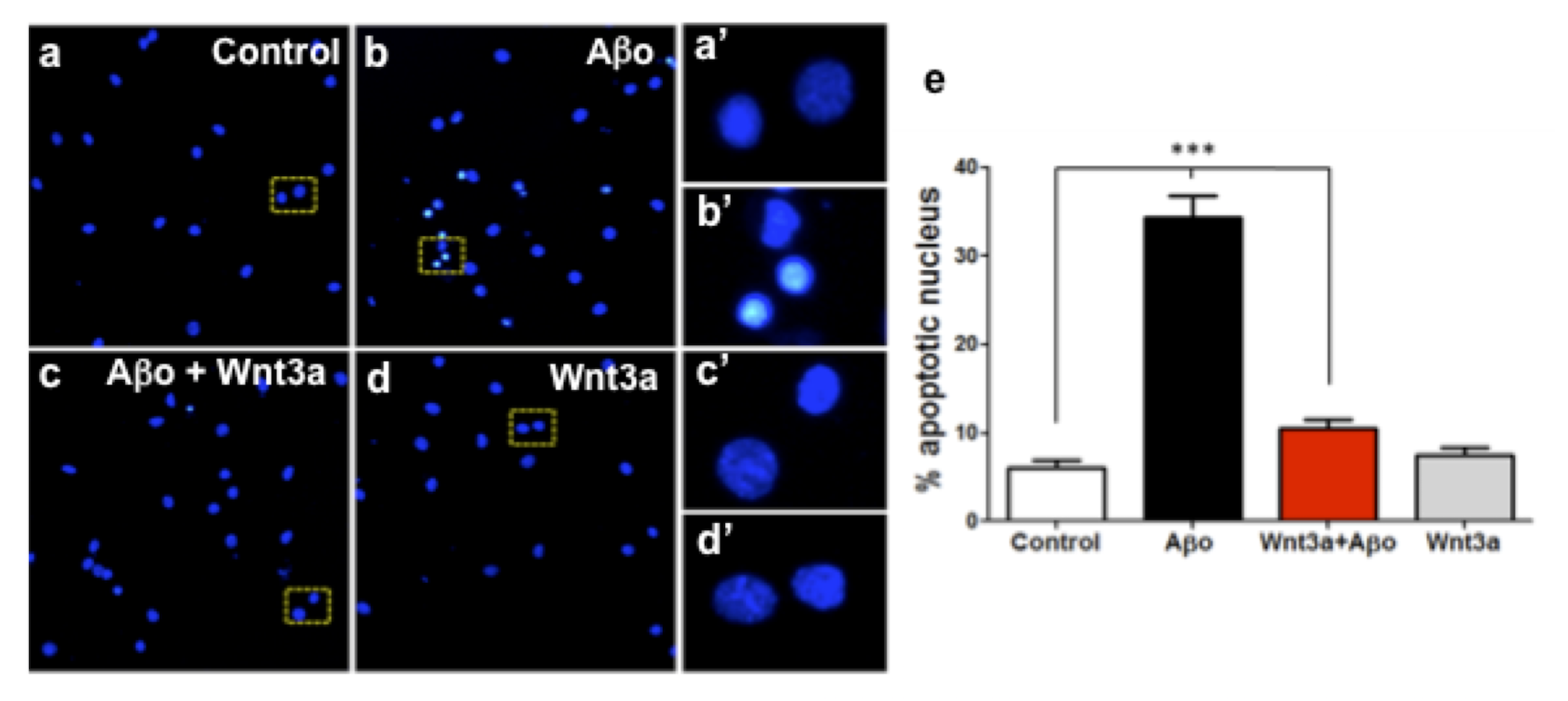

Supplement: S3 Fig — Neurons were co-incubated with Wnt3a protein and 5μM Aβo for 24 h. Apoptotic nuclei were detected with Hoechst stain (1μg/ml) in fixed neurons (a-d). Magnification shows representative nucleus of neurons treated with control media (a’), Aβo (b’), Wnt3a+Aβo (c’) and Wnt3a alone (d’). Graph shows the quantification of percentage of apoptotic nuclei in each condition (e). Statistical analysis in both experiments was carried out using one-way ANOVA test with a post hoc Bonferroni with ***p<0,0005. Quantifications represent the results of six independent experiments. (TIFF) [file pone.0168840.s015.tiff]
