# Supplementary material for: Wnt Signaling Prevents the Aβ Oligomer-Induced Mitochondrial Permeability Transition Pore Opening Preserving Mitochondrial Structure in Hippocampal Neurons
Source: PLoS One. 2017 Jan 6;12(1):e0168840. doi: 10.1371/journal.pone.0168840 (PMC5218554; doi:10.1371/journal.pone.0168840)
Supplement: S1 File — (DOCX) [file pone.0168840.s016.docx]

# Supporting Information

**Wnt signaling prevents the Aβ oligomer-induced mitochondrial permeability transition pore opening preserving mitochondrial structure and protecting hippocampal neurons**

# Macarena S. Arrázola^1^, Daniela Ordenes^1^ and Nibaldo C. Inestrosa^1,2,3*^

^1^Centro de Envejecimiento y Regeneración (CARE), Departamento de Biología Celular y Molecular, Facultad de Ciencias Biológicas, Pontificia Universidad Católica de Chile, Santiago, Chile, 8331150.

^2^Center for Healthy Brain Ageing, School of Psychiatry, Faculty of Medicine, University of New South Wales, Sydney, Australia.

^3^Centro de Excelencia en Biomedicina de Magallanes (CEBIMA), Universidad de Magallanes, Punta Arenas, Chile.

* Corresponding author: Dr. Nibaldo C. Inestrosa at CARE, Biomedical Research Center, Pontificia Universidad Católica de Chile, Av. Alameda 340, Santiago, Chile, postal code 8331150. Phone: + (56)-2-6862724; Fax: + (56)-2-6862959; E-mail: **[ninestrosa@bio.puc.cl](mailto:ninestrosa@bio.puc.cl)**

**Supplementary Materials and Methods**

**Primary culture of rat hippocampal neurons**

Sprague-Dawley rat hippocampal cultures were prepared as described previously [1]. Hippocampi from rats at embryonic day 18 were removed, dissected free of meninges in Ca^2+^/Mg^2+^-free Hanks’ balanced salt solution (HBSS). The tissue was resuspended in HBSS containing 0.25% (wt/vol) trypsin and incubated for 15 min at 37°C. After three rinses with HBSS, the tissue was mechanically dissociated in Dulbecco’s modified Eagle’s medium (GIBCO, Rockville, MD) supplemented with 10% horse serum (GIBCO), 100 U/ml penicillin and 100 µg/ml streptomycin. Dissociated hippocampal cells were seeded onto poly-L-lysine-coated wells in plating medium. Cultures were maintained at 37°C in 5% CO_2_ for 2 h before the plating medium was replaced with neurobasal growth medium (GIBCO) supplemented with B27 (GIBCO), 2 mM L-glutamine, 100 U/ml penicillin and 100 µg/ml streptomycin. On day 2, the cultured neurons were treated with 2 µM cytosine arabinoside (AraC) for 24 h.

**Electron microscopy**

Hippocampal slices were prepared as described previously [2,3]. Briefly, transverse slices (400 µm) from the dorsal hippocampus were cut under cold ACSF using a Vibroslice microtome (VSL, WPI) and incubated in oxygenated ACSF for more than 1 hour at room temperature before treatment. Hippocampal slices were treated for 4 h with Wnt3a plus 1 h of Aβos and were then immediately fixed in 3% glutaraldehyde in 50 mM cacodylate buffer (pH 7.2) for 3 days at room temperature. Next, the slices were treated for 90 min with 1% osmium tetroxide in cacodylate buffer, followed by treatment with 1% aqueous uranyl acetate, dehydration in acetone and embedding in Epon resin. The Epon-embedded lamina of the hippocampal slices was cut into small pieces and re-included in the resin. Areas to be examined by electron microscopy (CA1 region of the hippocampus) were selected from 1-µm sections stained with toluidine blue for light microscopy. Ultra-thin sections were cut with a Reichert Ultramicrotome, placed on 300-mesh copper electron microscopy grids, stained with uranyl acetate and lead citrate [4], and examined using a Phillips Tecnai 12 transmission electron microscope at 80 kV at the Electron Microscope Facility of the Faculty of Biological Sciences, Pontificia Universidad Católica de Chile, Santiago, Chile.

**Neuronal viability with Hoechst staining**

Hippocampal neurons plated on polylysine-coated coverslips (30,000 neurons/cover) were treated under different conditions for 24 h. Apoptotic nuclei were analyzed with Hoechst 33342 stain (1 μg/ml un distilled water) (Molecular Probes) in fixed cells after the treatments, as previously described [5,6]. Hoechst staining in hippocampal slices was developed as previously described for histochemical procedures [7,8]. Fixed slices were incubated for 5 min with 10 μg/ml Hoechst in distilled water and then washed and mounted in coverslips. Fluorescence intensity was analyzed in an epifluorescence microscope using and UV filter.

**References**

1. Caceres A, Banker G, Steward O, Binder L, Payne M. MAP2 is localized to the dendrites of hippocampal neurons which develop in culture. Brain Res. 1984;315: 314–8.

2. Cerpa W, Godoy J a, Alfaro I, Farías GG, Metcalfe MJ, Fuentealba R, et al. Wnt-7a modulates the synaptic vesicle cycle and synaptic transmission in hippocampal neurons. J Biol Chem. 2008;283: 5918–27. doi:10.1074/jbc.M705943200

3. Varela-Nallar L, Alfaro IE, Serrano FG, Parodi J, Inestrosa NC. Wingless-type family member 5A (Wnt-5a) stimulates synaptic differentiation and function of glutamatergic synapses. Proc Natl Acad Sci U S A. 2010;107: 21164–9. doi:10.1073/pnas.1010011107

4. Reynolds ES. The use of lead citrate at high pH as an electron-opaque stain in electron microscopy. J Cell Biol. 1963;17: 208–12.

5. Silva-Alvarez C, Arrázola MS, Godoy J a, Ordenes D, Inestrosa NC. Canonical Wnt signaling protects hippocampal neurons from Aβ oligomers: role of non-canonical Wnt-5a/Ca(2+) in mitochondrial dynamics. Front Cell Neurosci. 2013;7: 97. doi:10.3389/fncel.2013.00097

6. Godoy JA, Allard C, Arrázola MS, Zolezzi JM, Inestrosa NC. SIRT1 Protects Dendrites, Mitochondria and Synapses from Aβ Oligomers in Hippocampal Neurons. J Alzheimers Dis Park. 2013;03. doi:10.4172/2161-0460.1000126

7. Toledo EM, Inestrosa NC. Activation of Wnt signaling by lithium and rosiglitazone reduced spatial memory impairment and neurodegeneration in brains of an APPswe/PSEN1DeltaE9 mouse model of Alzheimer’s disease. Mol Psychiatry. Nature Publishing Group; 2010;15: 272–85, 228. doi:10.1038/mp.2009.72

8. Carvajal FJ, Zolezzi JM, Tapia-Rojas C, Godoy JA, Inestrosa NC. Tetrahydrohyperforin decreases cholinergic markers associated with amyloid-β plaques, 4-hydroxynonenal formation, and caspase-3 activation in AβPP/PS1 mice. J Alzheimers Dis. 2013;36: 99–118. doi:10.3233/JAD-130230
