# Supplementary material for: Wnt Signaling Prevents the Aβ Oligomer-Induced Mitochondrial Permeability Transition Pore Opening Preserving Mitochondrial Structure in Hippocampal Neurons
Source: PLoS One. 2017 Jan 6;12(1):e0168840. doi: 10.1371/journal.pone.0168840 (PMC5218554; doi:10.1371/journal.pone.0168840)
Supplement: S2 File — (DOC) [file pone.0168840.s017.doc]

# Supporting Information

**Wnt signaling prevents the Aβ oligomer-induced mitochondrial permeability transition pore opening preserving mitochondrial structure and protecting hippocampal neurons**

# Macarena S. Arrázola^1^, Eva Ramos-Fernández^1^, Pedro Cisternas^4^, Daniela Ordenes^1^ and Nibaldo C. Inestrosa^1,2,3*^

^1^Centro de Envejecimiento y Regeneración (CARE), Departamento de Biología Celular y Molecular, Facultad de Ciencias Biológicas, Pontificia Universidad Católica de Chile, Santiago, Chile, 8331150.

^2^Center for Healthy Brain Ageing, School of Psychiatry, Faculty of Medicine, University of New South Wales, Sydney, Australia.

^3^Centro de Excelencia en Biomedicina de Magallanes (CEBIMA), Universidad de Magallanes, Punta Arenas, Chile.

^4^ Universidad de Atacama, Facultad de Ciencias Naturales, Departamento de Química y Biología, Copayapu 485, Copiapó, Chile

* Corresponding author: Dr. Nibaldo C. Inestrosa at CARE, Biomedical Research Center, Pontificia Universidad Católica de Chile, Av. Alameda 340, Santiago, Chile, postal code 8331150. Phone: + (56)-2-6862724; Fax: + (56)-2-6862959; E-mail: [**ninestrosa@bio.puc.cl**](mailto:ninestrosa@bio.puc.cl)

**Western blot and Immunoprecipitation**

**Fig7 Original blots**


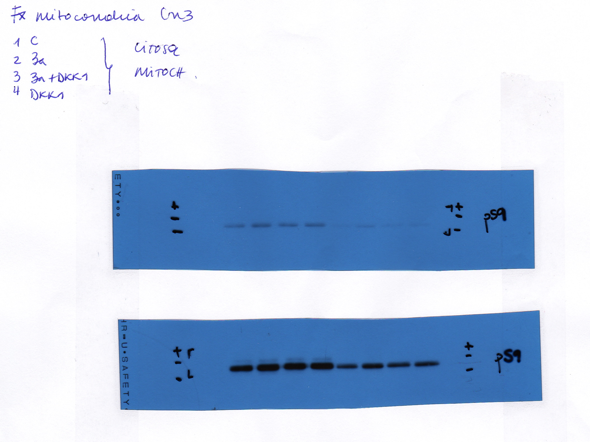

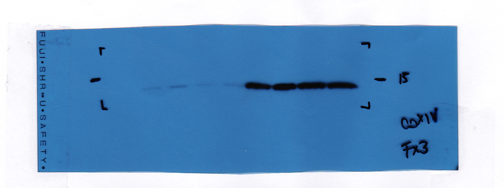


WB: p-GSK3β and COXIV as loading control of the mitochondrial fraction

**Fig7 Original blots from p-GSK3β immunoprecipitation**

Lane 1-4:input

Lane 5-8: IP p-GSK3β Ser9

Lane 9-12: supernatant from the IP

Control and Wnt3a treatment are shown in duplicate, as shows the inserted text in yellow

IP:p-GSK3β

WB: p-GSK3β


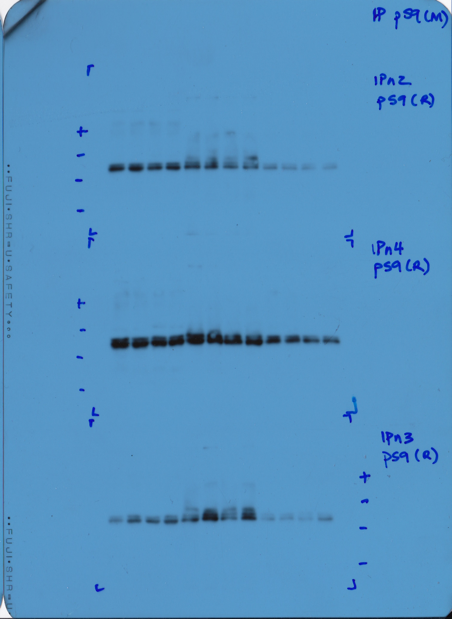


Input IP SN

C 3a C 3a C 3a C 3a C 3a C 3a

C 3a

C 3a

C 3a

IP:p-GSK3β

WB: ANT


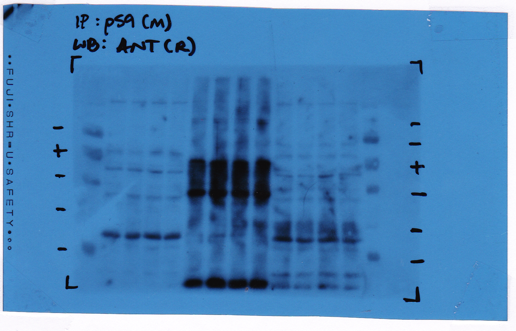


← ANT

IP:p-GSK3β

WB: CypD


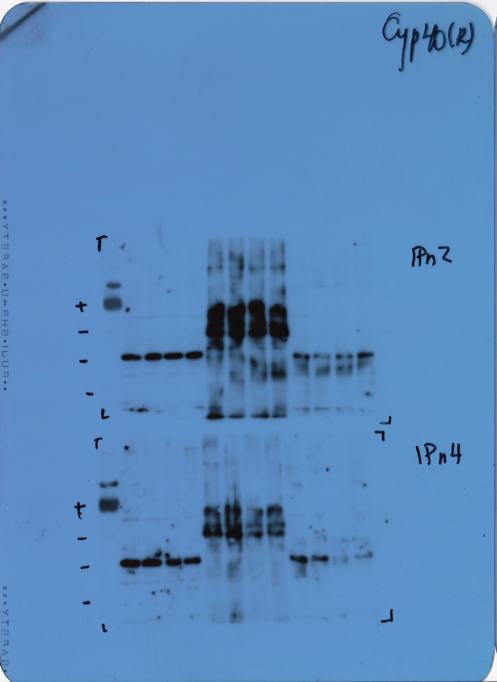


← CypD

IP:p-GSK3β

WB: total GSK3β and GAPDH


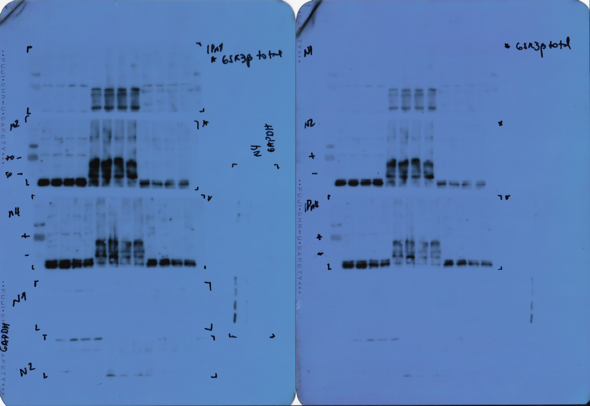


← GSK3b

← GAPDH

**Fig8 Original blots**

WB: HKII

(bands at the bottom correspond to a previous detection of p-GSK3β in the same membrane)


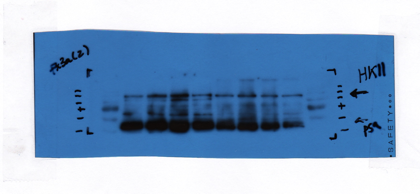


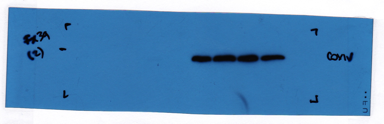
WB: COXIV
